# The Vitamin B_12_-Dependent Photoreceptor AerR Relieves Photosystem Gene Repression by Extending the Interaction of CrtJ with Photosystem Promoters

**DOI:** 10.1128/mBio.00261-17

**Published:** 2017-03-21

**Authors:** Mingxu Fang, Carl E. Bauer

**Affiliations:** Molecular and Cellular Biochemistry Department, Indiana University, Bloomington, Indiana, USA; University of Washington

**Keywords:** PpsR ortholog, global transcription factor, redox regulation, transcriptional regulation, transcriptomics, photoreceptor

## Abstract

Purple nonsulfur bacteria adapt their physiology to a wide variety of environmental conditions often through the control of transcription. One of the main transcription factors involved in controlling expression of the *Rhodobacter capsulatus* photosystem is CrtJ, which functions as an aerobic repressor of photosystem genes. Recently, we reported that a vitamin B_12_ binding antirepressor of CrtJ called AerR is required for anaerobic expression of the photosystem. However, the mechanism whereby AerR regulates CrtJ activity is unclear. In this study, we used a combination of next-generation sequencing and biochemical methods to globally identify genes under control of CrtJ and the role of AerR in controlling this regulation. Our results indicate that CrtJ has a much larger regulon than previously known, with a surprising regulatory function under both aerobic and anaerobic photosynthetic growth conditions. A combination of *in vivo* chromatin immunoprecipitation-DNA sequencing (ChIP-seq) and ChIP-seq and exonuclease digestion (ChIP-exo) studies and *in vitro* biochemical studies demonstrate that AerR forms a 1:2 complex with CrtJ (AerR-CrtJ_2_) and that this complex binds to many promoters under photosynthetic conditions. The results of *in vitro* and *in vivo* DNA binding studies indicate that AerR-CrtJ_2_ anaerobically forms an extended interaction with the bacteriochlorophyll *bchC* promoter to relieve repression by CrtJ. This is contrasted by aerobic growth conditions where CrtJ alone functions as an aerobic repressor of *bchC* expression. These results indicate that the DNA binding activity of CrtJ is modified by interacting with AerR in a redox-regulated manner and that this interaction alters CrtJ’s function.

## IMPORTANCE

Photoreceptors control a wide range of physiology often by regulating downstream gene expression in response to light absorption via a bound chromophore. Different photoreceptors are known to utilize a number of different compounds for light absorption, including the use of such compounds as flavins, linearized tetrapyrroles (bilins), and carotenoids. Recently, a novel class of photoreceptors that use vitamin B_12_ (cobalamin) as a blue-light-absorbing chromophore have been described. In this study, we analyzed the mechanism by which the vitamin B_12_ binding photoreceptor AerR controls the DNA binding activity of the photosystem regulator CrtJ. This study shows that a direct interaction between the vitamin B_12_ binding photoreceptor AerR with CrtJ modulates CrtJ binding to DNA and importantly, the regulatory outcome of gene expression, as shown here with photosystem promoters.

## INTRODUCTION

The metabolically versatile purple nonsulfur bacterium *Rhodobacter capsulatus* is capable of growth utilizing aerobic or anaerobic respiration, fermentation, or anoxygenic photosynthesis (1). When oxygen is plentiful, these cells primarily utilize oxidative phosphorylation, as this is one of the more efficient means of energy generation. However, when oxygen is limiting, these cells synthesize photosystems to capture and utilize solar energy for metabolic production (1).

As is the case in many species, *R. capsulatus* utilizes a number of redox-responding transcription factors to control gene expression in response to changes in oxygen levels. The transcription factors FnrL, RegA, and CrtJ appear to be the main regulators that *R. capsulatus* uses to control an aerobic-anaerobic metabolic switch (2–6). Among these three transcription factors, CrtJ (called PpsR in some species [7]) was thought to have the narrowest regulon and to play a role in aerobically repressing many photosystem genes such as *bch* (bacteriochlorophyll), *crt* (carotenoid), and *puc* (light-harvesting complex II) (6, 8). CrtJ is also known to aerobically repress genes coding for ubiquinol oxidase that has a respiratory role under conditions of low-oxygen tension (9). CrtJ is present in the genomes of all sequenced purple photosynthetic bacteria, typically in a cluster of genes involved in bacteriochlorophyll biosynthesis. In some species, there are two homologs present in the genome with one homolog involved in redox control and the other homolog regulated in response to light intensity via interaction with a photoreceptor (10, 11). The roles of CrtJ proteins are not the same in all purple bacterial species, as the CrtJ homolog from *Rubrivivax gelatinosus* is thought to be both a repressor and an activator (11).

Redox regulation of CrtJ has been mainly studied *in vitro* using the *bchC* promoter that contains two closely linked copies of a recognition palindrome sequence TGTN_12_ACA. One palindrome spans the −35 promoter recognition sequence, and the other palindrome spans the −10 promoter recognition sequences (12–14). Several *in vivo* and *in vitro* biochemical studies have demonstrated that CrtJ senses redox (mainly O_2_) via oxidation/reduction of redox-active cysteines. For example, cysteine 420 (C420) located in the DNA binding domain has been shown to form a disulfide bond with C249 (15, 16). C420 can also be oxidized to a stable sulfenic acid (Cys-SOH) derivative (15). *In vitro* DNA binding studies have shown that the binding affinity of CrtJ to the *bchC* promoter increases when C420 is oxidized, which promotes repression of expression (15).

In addition to redox control, there is also light control of CrtJ activity through its interaction with photoreceptors. In *Bradyrhizobium* sp. strain ORS278, *Rhodopseudomonas palustris*, and *Thiocapsa roseopersicina*, the CrtJ ortholog *ppsR2* is located upstream of a bacteriophytochrome-like photoreceptor BphP (4, 10). BphP is thought to interact and disrupt the binding of PpsR2 in a light-dependent manner with this system recently developed into an optogenetic tool (17). In *Rhodobacter sphaeroides*, the activity of the CrtJ homolog, PpsR, is also regulated by the light-absorbing antirepressor AppA that uses flavin as a blue-light-absorbing chromophore (18, 19). In *R. capsulatus*, it was recently shown that light also controls the activity of CrtJ via the blue-light-absorbing antirepressor AerR that uses vitamin B_12_ for blue-light absorption (20, 21). Loss of these photoreceptors results in severe PpsR/CrtJ-mediated reduction in photosystem synthesis, leading to a model in which these photoreceptors function as antirepressors (18, 20, 21). However, the ability of these photoreceptors to impede PpsR/CrtJ binding to target promoters has not been directly addressed *in vivo*.

Even though current models for redox and light regulation of CrtJ explain both the aerobic repressor role of CrtJ and the light-regulated antirepressor role of AerR, they are based primarily on *in vitro* studies with purified components and a limited set of DNA targets. These models lack support from *in vivo* DNA binding data that contain the additional complexities of buffered cellular redox and the interaction of CrtJ with additional proteins. In this study, we use next-generation sequencing methods such as transcriptome sequencing (RNA-seq), chromatin immunoprecipitation-DNA sequencing (ChIP-seq), and ChIP-exo to dissect the *in vivo* roles of CrtJ and AerR. Our results indicate that the number and variety of CrtJ target promoters are much more extensive than previously thought and that CrtJ regulates gene expression under both aerobic and anaerobic photosynthetic growth conditions. We further show that AerR has a more nuanced role than previously appreciated, as its main role is to function as a switch that alters CrtJ binding at target sequences to relieve repressor activity.

## RESULTS

### Transcriptome analysis reveals that CrtJ unexpectedly regulates expression aerobically and photosynthetically.

We explored the extent of the CrtJ regulon using a combination of RNA-seq, which measures genome-wide changes in gene expression (22, 23), and ChIP-seq, which measures the chromosome locations of affinity-tagged CrtJ *in vivo* (24). These transcriptome experiments were performed under both aerobic and anaerobic photosynthetic conditions.

Analysis of differentially expressed genes (DEGs) (derived from pairwise comparison of wild-type versus Δ*crtJ* RNA-seq data sets) shows that there are a large number of genes that exhibit reproducible changes in expression with a *P* value of ≤0.01 (see Tables S1 and S2 in the supplemental material). Under aerobic growth conditions (Table S1), the vast majority of DEGs (37 out of 54 total genes) are those involved in synthesis of the photosystem, such as the *bch* and *crt* genes that code for enzymes involved in bacteriochlorophyll and carotenoid synthesis, respectively, as well as the *puf* and *puc* genes that code for light-harvesting and reaction center structural components of the photosystem. The observed two- to four-fold increase in expression of these genes upon deletion of CrtJ is in agreement with prior studies which show that CrtJ functions as a modest aerobic repressor of *bch*, *crt*, and *puc* expression (8, 12, 15). In addition to components of the photosystem, CrtJ is also involved in aerobic activation of genes in the *cco* operon that code for components of *cbb*_*3*_-type cytochrome oxidase. Beyond photosynthesis and respiration, CrtJ is also involved in aerobic activation of components of a peptide nickel ABC transporter (*rcc00099* and *rcc00103*) and aerobic repression of a phosphotransferase system (PTS) fructose-specific transporter (*rcc02541* and *rcc02543*) and an aldehyde dehydrogenase (*rcc02015*).

10.1128/mBio.00261-17.5 TABLE S1  Differentially expressed genes in a Δ*crtJ* strain under aerobic condition as assayed by RNA-seq. Download TABLE S1, PDF file, 0.1 MB. Copyright © 2017 Fang and Bauer. 2017 Fang and Bauer This content is distributed under the terms of the Creative Commons Attribution 4.0 International license.

10.1128/mBio.00261-17.6 TABLE S2 Differentially expressed genes in a Δ*crtJ* strain under anaerobic photosynthetic conditions as assayed by RNA-seq. Download TABLE S2, PDF file, 1 MB. Copyright © 2017 Fang and Bauer. 2017 Fang and Bauer This content is distributed under the terms of the Creative Commons Attribution 4.0 International license.

As discussed above, several *in vitro* biochemical studies show that the DNA binding activity of CrtJ is stimulated under oxidizing conditions and reduced under reducing conditions (13, 15, 16). Deletion of *crtJ* also leads to increased aerobic synthesis of the photosystem *in vivo* (15). These results supported a model in which CrtJ functions as an aerobic repressor with little or no role under anaerobic photosynthetic conditions (15). Consequently, it is surprising that RNA-seq analysis shows that there are 84 genes that exhibit altered gene expression photosynthetically upon deletion of CrtJ (Table S2). Inspection of gene expression profiles that are photosynthetically affected by CrtJ shows that there is almost no overlap with those that are aerobically affected. The one notable exception is the linked *bchC-bchX* genes that code for enzymes in the bacteriochlorophyll biosynthetic pathway (Tables S1 and S2). Also of interest is CrtJ's involvement in the photosynthetic activation of *sufB* (*rcc01881*) that codes for an anaerobic FeS assembly protein. The bacteriochlorophyll *a* biosynthetic pathway codes for several enzymes such as dark protochlorphyllide reductase and chlorophyllide reductase that contain essential 4Fe4S centers, so anaerobic activation of *sufB* expression may be a consequence of the increased need to assemble 4Fe4S centers for these enzymes (25). CrtJ also photosynthetically activates a subunit of vitamin B_12_ binding methionine synthase (*rcc03352*) which is notable, as an early step in the bacteriochlorophyll biosynthetic pathway, Mg-protoporphyrin IX methyltransferase, utilizes *S*-adenosylmethionine as a cofactor (26, 27). Expression of several genes involved in motility such as genes coding for three methyl-accepting chemotaxis proteins (*rcc02611*, *rcc01667*, and *rcc02887*), *flgA* coding for a flagellar basal body P-ring assembly protein (*rcc03514*), and a *cheW* homolog (*rcc01356*) are also anaerobically repressed by CrtJ. Other notable anaerobically regulated genes include three transcription factors (*rcc01800*, *rcc00263*, and *rcc00602*) and an RNA polymerase sigma-32 (*rcc02811*) each of which is anaerobically repressed by CrtJ. There are also three diguanylate cyclase/phosphodiesterases (*rcc00620*, *rcc00645*, and *rcc02857*) that are photosynthetically activated by CrtJ (Table S2).

### ChIP-seq confirms that CrtJ targets genes under both aerobic and anaerobic photosynthetic conditions.

We analyzed the location of CrtJ binding to the genome *in vivo* by performing ChIP-seq analysis. For this analysis, we constructed both 3×FLAG-CrtJ epitope tag that was expressed from an isopropyl-β-d-thiogalactopyranoside (IPTG)-induced plasmid gene-encoded promoter and a 3×V5-CrtJ epitope that was recombined into the native chromosomal location with expression driven by the native CrtJ promoter. Functional complementation of these constructs, as well as analysis of CrtJ expression levels, is provided in Text S2. ChIP-seq peaks were designated as being meaningful only if they were considered significantly above background by the *m*odel-based *a*nalysis of *C*hIP-*S*eq program (MACS) in at least three of five independent biological replicates and also present in both the 3×FLAG-CrtJ and 3×V5-CrtJ data sets.

**FIG 1:**
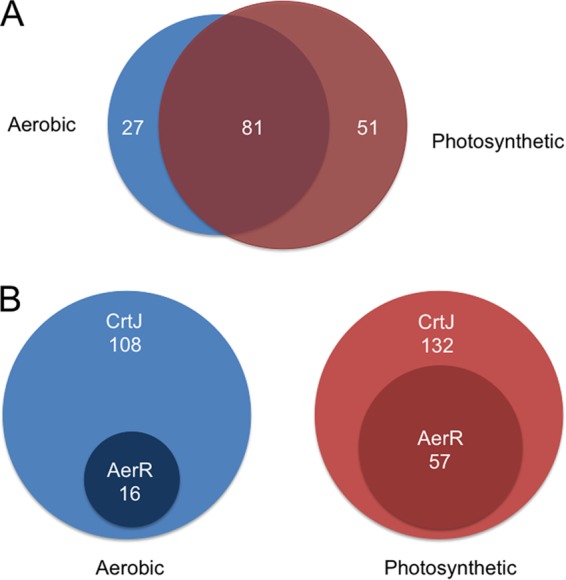
Venn diagram of the numbers of called CrtJ and AerR ChIP-seq peaks under different growth conditions. (A) CrtJ ChIP-seq peaks observed under aerobic (blue) and photosynthetic (brown) conditions exhibit extensive, but not total, overlap. (B) AerR ChIP-seq peaks are present at the same location as CrtJ in both aerobic and anaerobic photosynthetic conditions with many more overlapping sites photosynthetically than aerobically.

**FIG 2:**
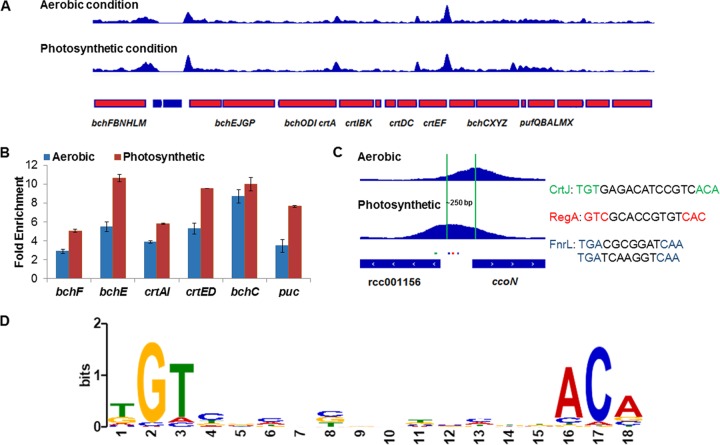
ChIP-seq analysis of CrtJ binding to photosystem and respiratory promoters. (A) ChIP-seq peaks obtained at numerous locations in the photosynthesis gene cluster (genes in red) under aerobic and photosynthetic growth conditions. (B) ChIP fold enrichment at various photosynthesis gene promoters under aerobic and anaerobic photosynthetic conditions. (C) Different CrtJ ChIP-seq peak summits at the *ccoN* promoter under aerobic versus photosynthetic conditions. Green, red, and blue dots indicate binding sequence for CrtJ, RegA, and FnrL, respectively. (D) Degenerate consensus sequence present in peaks bound by CrtJ under both aerobic and anaerobic photosynthetic conditions. Details of the method used to generate this consensus sequence are covered in Materials and Methods with a list of ChIP peaks that contain this sequence present in Tables S1 and S2 in the supplemental material.

Under aerobic growth conditions, a total of 108 ChIP-seq peaks were considered significant (Fig. 1A; Table S3). Of these 108 peaks, 7 peaks could be assigned to genes that undergo differential expression upon deletion of *crtJ* as assayed by RNA-seq (Table S1). Peaks were called within the *crtAI*, *bchC*, and *puc* promoters with peak summits that were congruent with previously observed CrtJ binding to its TGTN_12_ACA consensus recognition sequences as assayed by DNase I footprint protection (13, 15). There were also peaks called upstream of the *bchF*, *bchE*, and *crtED* promoters that also contain this CrtJ binding consensus sequence (Fig. 2A). These promoter regions comprise components of a photosynthesis superoperon that collectively control the expression of ~40 photosynthetic genes that are known to be aerobically repressed by CrtJ (13). These ChIP-seq and RNA-seq results also are in agreement with previous results derived from β-galactosidase and quantitative PCR analyses (8, 15, 28).

10.1128/mBio.00261-17.7 TABLE S3  Called CrtJ ChIP-Seq peaks under aerobic conditions. Download TABLE S3, PDF file, 1.7 MB. Copyright © 2017 Fang and Bauer. 2017 Fang and Bauer This content is distributed under the terms of the Creative Commons Attribution 4.0 International license.

In addition to the numerous called CrtJ binding sites among photosynthesis genes, there is also a called binding peak upstream of the *ccoNOQP* operon that codes for *cbb*_*3*_-type cytochrome oxidase (Fig. 2C). As discussed above, RNA-seq analysis indicates that CrtJ is functioning as an aerobic activator of this operon. Finally, there are 101 called peaks for which we do not observe changes in neighboring gene expression. Similar ChIP-seq-identified “silent” binding sites have been reported for several other transcription factors such as cyclic AMP receptor protein (CRP), FnrL, fumarate and nitrate reductase (FNR), LuxR, and CgrA (5, 29–32). These silent peaks imply additional potential regulation by CrtJ that presumably do not show regulation under the assayed RNA-seq conditions due to other transcription factors that are dominant.

Under anaerobic photosynthetic conditions, there were 132 called peaks (Fig. 1A; Table S4). Interestingly, 81 peaks out of a total of 159 peaks (aerobic plus anaerobic photosynthetic peaks) are present in similar locations under both growth conditions with 27 peaks unique to aerobic conditions and 51 unique to photosynthetic conditions (Fig. 1A; Table S5). CrtJ peaks upstream of the photosystem genes *bchF*, *bchE*, *crtED*, *crtAI*, *bchC*, and *puc* were present at comparable fold enrichments under aerobic and anaerobic photosynthetic conditions, if not slightly higher under the photosynthetic condition (Fig. 2B). Also of note are changes in the locations of peak summits as well as alterations in peak heights in the *ccoN* promoter, which exhibits an ~250-bp peak summit switch (Fig. 2C). Since both FnrL and RegA have binding sites on this promoter, the peak summit shift may imply control of *ccoN* transcription by one of these three transcription factors under different conditions.

10.1128/mBio.00261-17.8 TABLE S4 Called CrtJ ChIP-Seq peaks under anaerobic photosynthetic conditions. Download TABLE S4, PDF file, 1 MB. Copyright © 2017 Fang and Bauer. 2017 Fang and Bauer This content is distributed under the terms of the Creative Commons Attribution 4.0 International license.

10.1128/mBio.00261-17.9 TABLE S5 Unique and common CrtJ binding sites under aerobic and anaerobic photosynthetic growth conditions. Download TABLE S5, PDF file, 0.1 MB. Copyright © 2017 Fang and Bauer. 2017 Fang and Bauer This content is distributed under the terms of the Creative Commons Attribution 4.0 International license.

We also analyzed the presence of a conserved recognition sequence within the called 159 peaks by analyzing for sequence conservation within 200 bp relative to the peak summit using MEME (33). A binding motif of tGTN_12_ACa (Fig. 2D) was identified by this algorithm that exhibits a close fit to the TGTN_12_ACA sequence derived from previous DNase I footprint analysis (12–14).

### ChIP-exo reveals altered aerobic versus photosynthetic CrtJ binding to the *bchC* promoter.

Prior *in vitro* DNase I footprint studies have shown that CrtJ binds to two palindromes in the *bchC* promoter that have the conserved TGTN_12_ACA CrtJ recognition sequence (13, 14, 20). The “lower” recognition palindrome is present from positions −4 to −21, and the second “upper” palindrome is present from positions −30 to −47 relative to the start of transcription (13, 14, 20). The lower palindrome thus spans the −10 promoter recognition sequence, while the upper palindrome spans the −35 recognition sequence.

**FIG 3:**
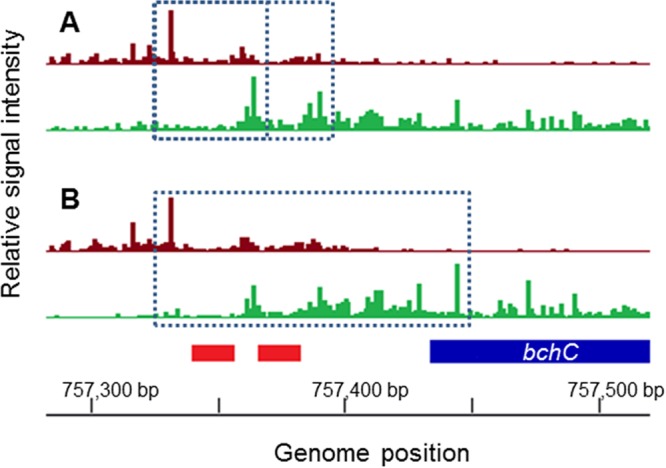
*In vivo* ChIP-exo analysis of CrtJ binding borders on the *bchC* promoter under aerobic and anaerobic photosynthetic growth conditions. (A) Plus-strand (magenta) and minus-strand (green) borders of the *bchC* promoter revealed by ChIP-exo under aerobic conditions. (B) Plus- and minus-strand borders as described above under photosynthetic growth conditions. The two red boxes represent the locations of the two CrtJ recognition sites with the blue box representing the *bchC* coding region.

To address the nature of CrtJ binding to the *bchC* promoter *in vivo* at a similar fine resolution, we used the recently described ChIP-exo procedure. The ChIP-exo procedure incorporates an additional 5′-to-3′ exonuclease digestion step into ChIP-seq just prior to reverse cross-linking and sequence analysis (34, 35). Data from merged independent replicate ChIP-exo experiments detect borders of transcription factor binding sites at nucleotide resolution as processed by MACE software (36). These transcription factor binding site borders are indicated as sequencing reads that start with a particular nucleotide at the highest frequency. The *in vivo* ChIP-exo results in Fig. 3 show that CrtJ binds to the *bchC* promoter differently under aerobic and anaerobic photosynthetic grown conditions. In both aerobically and photosynthetically grown cells, there is a prominent upper strand left side border (as indicated by the largest magenta bar) that is 8 nucleotides upstream of the upper TGTN_12_ACA palindrome that spans the −35 promoter region (the locations of the recognition palindromes are indicated as red boxes below the number of ChIP-exo sequencing reads). However, the lower strand right-hand borders are distinctly different between aerobically and photosynthetically grown cells. In aerobically grown cells, there is a prominent right border located between the upper and lower TGTN_12_ACA palindromes and a second slightly less prominent right border 8 nucleotides downstream of the lower TGTN_12_ACA palindrome (Fig. 3A). We interpret these results as evidence that CrtJ indeed occupies both palindromes aerobically with greater occupation at the upper palindrome. This *in vivo* result correlates well with the results of *in vitro* CrtJ binding studies, which show that the upper palindrome has a higher affinity for CrtJ than does the lower palindrome (50% effective concentration [EC_50_] of 2.9 × 10^−9^ M versus 4.8 × 10^−9^ M, respectively) (13). However, in the case of photosynthetically grown cells, the most prominent right-hand border is located 54 nucleotides downstream (at position +58 relative to the start of transcription) (Fig. 3B). This result indicates that CrtJ exhibits significantly altered, and extended, binding to the *bchC* promoter region *in vivo* under photosynthetic conditions relative to aerobic conditions.

### AerR alters CrtJ binding *in vivo* and *in vitro.*

**FIG 4:**
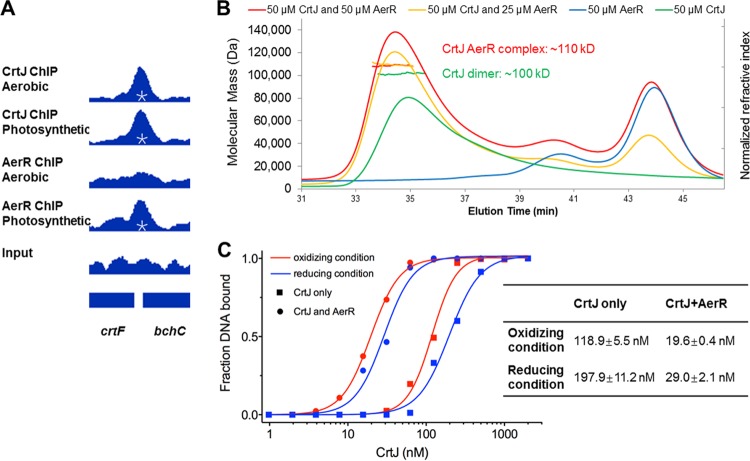
AerR increases the affinity of CrtJ to the *bchC* promoter by forming a complex with CrtJ. (A) ChIP-seq pulldown results with FLAG-tagged CrtJ showing that CrtJ localizes on the *bchC* promoter under both aerobic and photosynthetic growth conditions. A similar ChIP-seq study with FLAG-tagged AerR shows that AerR colocalizes with CrtJ to the *bchC* promoter under photosynthetic, but not aerobic, conditions. Reads obtained on a control reaction that did not undergo immunoprecipitation (labeled Input) are shown at the bottom of the panel. Peaks with an embedded asterisk are the peaks that were called significant by MACS (model-based analysis of ChIP-Seq). (B) Multiangle light scattering analysis of CrtJ alone, which exhibits an average molecular mass of ~100 kDa (green line), and of the AerR-CrtJ complex with an average molecular mass of ~110 kDa (yellow and red lines). (C) Binding isotherms of CrtJ to the *bchC* promoter under different conditions as generated from gel mobility shift assays. The addition of AerR increased the affinity of CrtJ to the promoter under both oxidizing (O_2_) and reducing (10 mM DTT) conditions. The table to the right of the graph shows the concentration of CrtJ at which 50% of the DNA probe was shifted (50% effective concentration [EC_50_]) when fitted to the Hill equation.

In all sequenced purple bacterial species, *crtJ* is colocalized in the genome with its coregulator AerR (in some species, AerR is called PpaA) (21). It has been shown that AerR uses vitamin B_12_ as a cofactor and that AerR can affect the DNA binding activity of CrtJ (20, 21). In *R. capsulatus*, disruption of AerR results in constitutive repression of photosystem gene expression with this repression relieved in an *aerR crtJ* double deletion strain (20, 37). AerR is also known to physically interact with CrtJ *in vitro* (20). Because of this interaction, we investigated whether AerR affects the nature of CrtJ binding to the chromosome *in vivo* using several approaches. First, we addressed whether there is colocalization of AerR with CrtJ *in vivo* using ChIP-seq experiments with FLAG-tagged AerR under aerobic and anaerobic photosynthetic growth conditions. This analysis showed called AerR binding peaks at 16 locations under aerobic conditions and 57 locations under photosynthetic conditions (Fig. 1B; Tables S1 and S2). In each case, AerR ChIP-seq peaks colocalized with CrtJ ChIP-seq peaks (Fig. 1). A previous study demonstrated that AerR functions as a light receptor using vitamin B_12_ as a chromophore (20), so it is not surprising to see AerR has more binding sites under light photosynthetic conditions than dark aerobic conditions. Interestingly, AerR colocalizes with CrtJ at all previously characterized photosynthesis gene promoters under photosynthetic condition, but not under aerobic conditions. An example of AerR-CrtJ colocalization to the *bchC* promoter under photosynthetic conditions is shown in Fig. 4A.

We next addressed the nature of the interaction of AerR with CrtJ using multiangle light scattering (MALS), which can accurately determine the molecular weight of an AerR-CrtJ complex. This analysis (Fig. 4B) showed that the average molecular mass (MM) of a CrtJ-only peak was 101.5 kDa (standard deviation [SD], 0.6 kDa), which corresponded to the molecular mass of a CrtJ dimer that has a calculated MM of 101.0 kDa. When adding AerR with a calculated MM of 28.2 kDa, the elution time of CrtJ peak shifted, with the average molecular mass of these particles increasing to 109.5 kDa (SD, 0.5 kDa). This indicates that AerR likely forms a 1:2 complex with the CrtJ dimer (AerR-CrtJ_2_).

We next investigated the role of AerR in regulating the DNA binding activity of CrtJ by performing gel mobility shift assays with CrtJ alone as well as with the AerR-CrtJ_2_ complex. For this analysis, we used a 325-bp *bchC* DNA probe that contained the two tandem CrtJ binding sites in the *bchC* promoter region as well as 137 bp of the *bchC* coding region. These assays were performed under oxidizing condition or reducing conditions (10 mM dithiothreitol [DTT]). A binding isotherm of CrtJ_2_ and of AerR-CrtJ_2_ binding to the DNA probe shows that CrtJ_2_ had higher affinity to the *bchC* promoter under oxidizing conditions than under reducing conditions, which confirms prior DNA binding studies (Fig. 4C; Fig. S2) (13, 15, 16). Interestingly, the AerR-CrtJ_2_ complex exhibited significantly higher binding affinity (~6-fold) than CrtJ alone under both oxidizing and reducing conditions.

10.1128/mBio.00261-17.3 FIG S1  Results of DNase I footprint analysis of CrtJ_2_ and AerR-CrtJ_2_ binding to the *bchC* promoter. Download FIG S1, PDF file, 0.2 MB. Copyright © 2017 Fang and Bauer. 2017 Fang and Bauer This content is distributed under the terms of the Creative Commons Attribution 4.0 International license.

10.1128/mBio.00261-17.4 FIG S2  Results of gel mobility shift assays of CrtJ and AerR binding to the *bchC* promoter region under both oxidizing and reducing conditions. Download FIG S2, PDF file, 0.5 MB. Copyright © 2017 Fang and Bauer. 2017 Fang and Bauer This content is distributed under the terms of the Creative Commons Attribution 4.0 International license.

We also addressed the nature of binding of the AerR-CrtJ_2_ complex to the *bchC* promoter by undertaking DNase I footprint protection studies in the presence of CrtJ_2_ and in the presence of AerR-CrtJ_2_. The results of this *in vitro* analysis (Fig. S1) show that CrtJ_2_ alone exhibits an excellent protection site that spans the two TGTN_12_ACA palindromes (yellow bar in Fig. S1). However, a similar analysis with AerR-CrtJ_2_ shows that numerous nucleotides upstream (~20 bp upstream) of the upper recognition sequence became hypersensitive to DNase I digestion. Furthermore, there is additional downstream protection and several hypersensitive sites extending ~100 bp within the *bchC* coding region.

**FIG 5:**
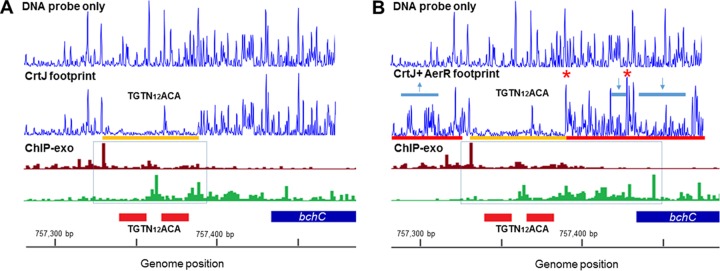
Overlay of DNase I footprint results and ChIP-exo results on the *bchC* promoter. Protection of CrtJ on the *bchC* promoter can be seen in both footprint and ChIP-exo results. (A and B) Under aerobic conditions, the *in vivo* binding of CrtJ is mainly in the two palindromes (A); however, under photosynthetic conditions, the *in vivo* protection, as well as the footprint with CrtJ and AerR, were extended (B). Combining the results, it can be inferred that under photosynthetic conditions, AerR can help CrtJ extend its binding on the *bchC* promoter.

Overlaying the *in vitro* DNase I protection patterns with the *in vivo* ChIP-exo results indicates that the CrtJ_2_ only footprint results correlates well with the aerobic ChIP-exo protection pattern, which shows borders flanking the two CrtJ recognition palindromes (Fig. 5). This result is also supported by our AerR ChIP-seq results, which show no binding of AerR to the *bchC* promoter regions under aerobic conditions. However, under anaerobic photosynthetic conditions, the extended downstream border observed with the *in vivo* ChIP-exo results shows excellent correlation with the extended *in vitro* DNase I protection pattern observed with the AerR-CrtJ_2_ complex. This result indicates that anaerobic photosynthetic formation of the AerR-CrtJ_2_ complex likely promotes enhanced and extended binding of CrtJ to the *bchC* promoter region.

**FIG 6:**
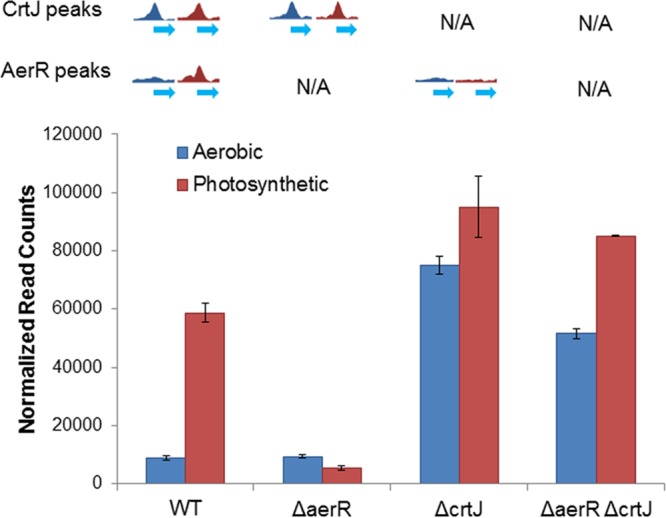
Comparative analysis of CrtJ and AerR chromosome occupancy relative to *bchC* expression. (Top) ChIP-seq occupancy of CrtJ and AerR to the *bchC* promoter region under aerobic conditions (blue peaks) and photosynthetic conditions (red peaks). The peaks of some strains were not available (N/A), as either CrtJ or AerR was deleted. (Bottom) *bchC* expression levels relative to CrtJ and AerR occupancy under aerobic and anaerobic photosynthetic conditions represented as normalized read counts derived from RNA-seq results. WT, wild type.

Finally, we analyzed *bchC* expression under aerobic and anaerobic photosynthetic conditions in wild-type cells, cells in which *aerR* was deleted, and cells in which either *crtJ* or *aerR* were deleted. Under aerobic conditions (Fig. 6, blue bars) where there is a prominent CrtJ ChIP-seq peaks centered on the *bchC* promoter region, *bchC* expression is low in the parent wild-type strain and in the strain where *aerR* was deleted. In these strains, CrtJ is present and presumably functioning as an aerobic repressor. This conclusion is supported by a sixfold increase in aerobic *bchC* expression exhibited by a *crtJ* deletion strain and an *aerR crtJ* double deletion strain. However, under photosynthetic conditions where both CrtJ and AerR cooccupy the *bchC* promoter region as measured by ChIP-seq, the expression level of *bchC* increased significantly in wild-type cells. In contrast, in the Δ*aerR* strain where CrtJ still occupies the *bchC* promoter region, there was no activation of *bchC* observed under photosynthetic conditions, indicating that AerR is necessary to allow *bchC* gene expression in the presence of CrtJ binding. In the Δ*crtJ* strain and in the Δ*aerR* Δ*crtJ* strain, the expression of *bchC* is high under both conditions. We interpret these results as evidence for aerobic repression by CrtJ alone and inactivation of CrtJ repression of *bchC* expression by AerR under anaerobic photosynthetic conditions.

## DISCUSSION

Prior to this study, *R. capsulatus* genes targeted by CrtJ were thought to primarily consist of those involved in synthesis of the photosystem and respiratory cytochromes. Specifically, genes involved in synthesis of bacteriochlorophyll and carotenoids and the light-harvesting apoproteins, as well as genes coding for a respiratory ubiquinol oxidase, were known targets of CrtJ (8, 9). The RNA-seq and ChIP-seq results in this study, which show that CrtJ directly aerobically represses a number of genes involved in photosynthesis and respiration (see Tables S1 and S3 in the supplemental material), are validated by these results. In addition, these data also provides many new CrtJ targets such as several genes involved in motility.

Previously two ChIP studies have been performed on PpsR, a CrtJ homolog in *R. sphaeroides*. Bruscella et al. (38) used quantitative reverse transcription-PCR (qRT-PCR) to analyze the ChIP-enriched DNA and found enrichment of *ccoN* and *ppaA* (*aerR* homolog in *R. sphaeroides*) promoters under aerobic conditions, which could also be found in our ChIP-seq result in *R. capsulatus*. The authors found that PpsR showed strong binding only under aerobic conditions, but not under anaerobic fermentative growth conditions. In our study, we found that CrtJ bound to the *ccoN* promoter under both aerobic and photosynthetic growth conditions. Bruscella et al. (38) also showed that in an PrrA^−^ (RegA homolog) strain, further disruption of PpsR would lead to differential expression of MetH and MetF homologs, but not in the strain with a PpsR disruption only. In our ChIP-seq study, we could clearly identify a peak for the *metH1* promoter, but no change in gene expression was seen, which suggests that RegA could be playing a role in this regulation. Imam et al. (39) also used ChIP-seq to identify PpsR peaks and found similar peaks as Bruscella et al. did (38). However, they found fewer genes to be controlled by PpsR comparing the expression profiles for the Δ*ppsR* strain and its parent strain. Since Bruscella et al. identified genes under control of PpsR by comparing the PpsR^−^ PrrA^−^ strain with the PrrA^−^ strain, the differences could be explained by PrrA blocking PpsR from regulating these genes. This finding can explain some of the silent peaks that were found in our ChIP-seq results, as many promoters regulated by CrtJ are also known to be regulated by RegA (Tables S1 and S2) (40).

One surprising result from our transcriptomic study is that deleting CrtJ has a significant effect on anaerobic photosynthetic gene expression (Table S2). Prior studies of CrtJ were focused on aerobic repression by CrtJ, so its role on photosynthetic gene expression has been largely unstudied. One reason why a photosynthetic role may have been overlooked is that deletion of CrtJ has little effect on photosynthetic expression of photosynthesis genes, which is a dominant visible (via pigmentation) physiological change that is exhibited by these cells. Furthermore, deletion of CrtJ leads to a well-characterized aerobic increase in photosystem gene expression, hence why CrtJ has until now been predominantly considered a dedicated aerobic photosystem repressor. Genes that are affected photosynthetically by CrtJ are largely distinct from those identified in aerobic studies. Inspection of Table S2 shows that a number of “genes of unknown function” increase expression, while a number of motility genes and genes of surprisingly varied functions undergo a decrease in anaerobic photosynthetic expression upon deletion of CrtJ.

Analysis of CrtJ binding sites *in vivo* using ChIP-seq also surprisingly reveal that CrtJ is bound to many targets under both aerobic and anaerobic photosynthetic conditions. This is different from prior *in vitro* studies, which indicated that the DNA binding activity of CrtJ is redox responsive. For example, several studies have demonstrated that CrtJ exhibits tighter binding to the *bchC* and *crtA-crtI* promoter regions under oxidizing conditions than under reducing conditions (12, 13). Previous studies also indicated that the redox state of Cys420 affects the DNA binding activity of CrtJ (15, 16). For example, *in vitro* DNA binding assays with purified CrtJ shows that oxidation of Cys420 promotes higher-affinity binding of CrtJ to the *bchC* promoter than does reduced CrtJ. These results were supported by several *in vivo* studies which also showed that Cys420 is oxidized to a mixture of a disulfide bond and a sulfenic acid in cells grown under aerobic growth conditions (15, 16). Collectively, these results lead to a redox model where aerobic oxidation of Cys420 stimulated the binding of CrtJ to target promoters, which in the case of photosystem genes resulted in repression.

Clearly, our study indicates that CrtJ binding to the *bch* promoter and other promoters is considerably more complex than the simplified redox-based model. This conclusion is supported by our observation that CrtJ binds to some targets under anaerobic photosynthetic conditions, binds to some targets under aerobic conditions, and binds to a majority of targets under both aerobic and anaerobic photosynthetic conditions. Interestingly, we also observed that AerR colocalizes with CrtJ to a majority of CrtJ binding sites under photosynthetic conditions, suggesting that CrtJ and AerR primarily (but not exclusively) function as coregulators under this condition.

**FIG 7:**
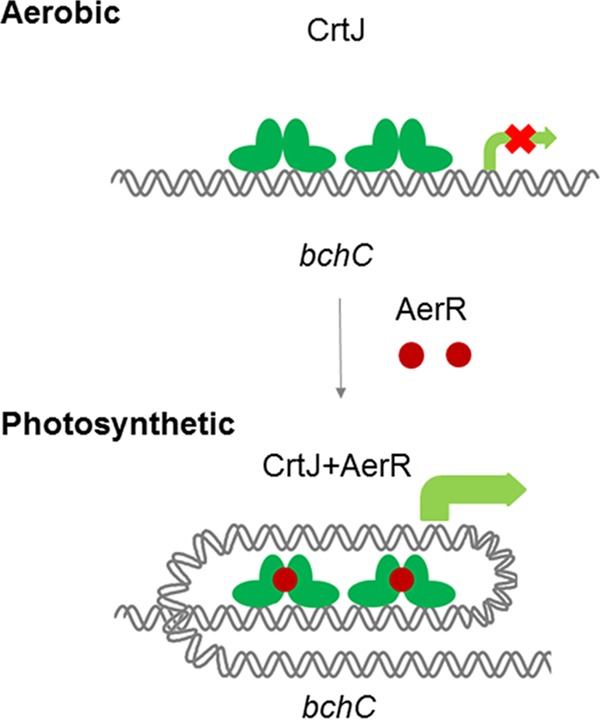
Model of CrtJ and AerR repression and activation of photosynthesis gene expression. Under aerobic growth conditions, CrtJ binds to recognition palindromes present in the promoters of photosynthesis genes, causing repression of gene expression. Under photosynthetic conditions, AerR forms a complex with CrtJ on photosystem promoters that promotes an extended interaction with the promoter region represented in our model as a loop that promotes activation of gene expression.

What is the mechanism of photosynthetic coregulation by CrtJ and AerR? Our ChIP-exo analysis shows a more extensive CrtJ binding pattern to the *bchC* promoter region under anaerobic photosynthetic repressing conditions than under aerobic activating conditions. These *in vivo* ChIP-exo results are mimicked by *in vitro* footprint assays which show that the AerR-CrtJ_2_ complex exhibits a similar highly extended footprint pattern relative to that observed with CrtJ alone. Gel mobility shift assays with DNA segments that extended into the *bchC* coding region further show that the AerR-CrtJ_2_ complex is able to bind to the *bchC* promoter region *in vitro* and that AerR actually increases the affinity of CrtJ to this region. Taken together, we can propose a revised model for CrtJ regulation of photosystem gene expression in which oxidized CrtJ is capable of binding to the *bchC* promoter alone under aerobic conditions leading to repression of expression (Fig. 7). However, we propose that under anaerobic reducing conditions, CrtJ forms an AerR-CrtJ_2_ complex that exhibits altered extended binding to the *bchC* promoter region, which appears to negate repression by CrtJ. One possibility is that this extended binding distorts the −35 and −10 region to make it more accessible for RNA polymerase. A similar mechanism was seen in MerR family transcription factors that can upregulate transcription by reconfiguring the −35 and −10 elements upon binding of a cofactor (41). Finally, it is not yet clear is why an AerR-CrtJ colocalization mechanism is a preferable mechanism of repressor control than simple release of CrtJ from the target DNA. Presumably, this mechanism of control provides a more refined and rapid mechanism of gene expression control than simple release of CrtJ from DNA targets.

The results of this study not only raise additional questions regarding the transcription control by CrtJ in *R. capsulatus* but also by its homolog PpsR from *R. sphaeroides*. For example, the activity of AerR is known to be activated by light-dependent binding of cobalamin (vitamin B_12_) (15). Specifically, a reduction in the level of vitamin B_12_ in cells leads to constitutive CrtJ-mediated repression of photosystem gene expression (15, 42, 43). At this time, it is unclear if AerR in the absence of B_12_ may retain the ability of positioning CrtJ to a different subset of locations relative to AerR with bound vitamin B_12_. Interestingly, a deletion of the AerR homolog (PpaA) in *R. sphaeroides* has little effect on synthesis of the photosystem (15, 37). This is distinctly different from the constitutive CrtJ-mediated repression of photosystem synthesis that occurs upon deletion of AerR in *R. capsulatus* (15, 37). What then regulates the DNA binding activity of PpsR to *R. sphaeroides* photosystem promoters? In *R. sphaeroides*, there is an additional photoreceptor regulator of PpsR called AppA that has a heme binding domain (SCHIC [*s*ensor *c*ontaining *h*eme *i*nstead of *c*obalamin] domain) that exhibits significant similarity with the vitamin B_12_ binding domain of AerR/PpaA (41, 44–46). Furthermore, AppA has a flavin binding photoreceptor domain (BLUF [*b*lue *l*ight *u*sing *F*AD {flavin adenine dinucleotide} domain) (19) that regulates the binding of AppA to PpsR in a light-regulated manner (light-excited AppA does not bind PpsR) (18). Deletion of AppA in *R. sphaeroides* leads to constitutive PpsR-mediated repression of photosystem gene expression similar to that observed upon deletion of AerR in *R. capsulatus* (47). This suggests that AppA may be functioning as a master regulator of PpsR activity at photosystem promoters. Furthermore, it has been shown that AppA can also form a 2:1 complex with PpsR, not unlike that described for AerR and CrtJ, and that this complex can bind to large DNA segments (48). Consequently, it is likely that the DNA binding activity of *R. sphaeroides* PpsR is regulated by two related photoreceptors, PpaA that binds vitamin B_12_ in a light-dependent manner and AppA that binds heme and is regulated by light absorption via a bound flavin. To date, the role of PpaA in controlling the activity of PpsR remains unclear, but it is possible that PpaA and AppA together coordinate the binding of PpsR to different subsets of promoters. Additional *in vivo* studies with PpsR in the presence and absence of AppA and PpaA, as well as the localization of the two proteins on the genome, will have to be undertaken to determine if this is indeed the case.

In conclusion, we have applied a combination of next-generation sequencing and biochemical method to study CrtJ regulation in *R. capsulatus*. On the basis of results from RNA-seq and CrtJ ChIP-seq, we have expanded the CrtJ regulon. ChIP-exo analyses also show that CrtJ exhibits altered binding to the *bchC* promoter under aerobic and anaerobic photosynthetic growth conditions. Finally, ChIP-seq and DNase footprint assays show that AerR colocalized with CrtJ to the *bchC* promoter *in vivo* and alter its binding on the promoter *in vitro*. Elucidating the molecular mechanism of the AerR-CrtJ system provides not only a new mechanism of regulating bacterial physiology but also additional understanding of the light-sensing tools used to regulate transcription in this organism and in other systems.

## MATERIALS AND METHODS

For more-detailed methodologies, see Text S1 in the supplemental material.

10.1128/mBio.00261-17.1 TEXT S1  Supplemental Materials and Methods. Download TEXT S1, PDF file, 9.2 MB. Copyright © 2017 Fang and Bauer. 2017 Fang and Bauer This content is distributed under the terms of the Creative Commons Attribution 4.0 International license.

### Strains and growth conditions.

*Rhodobacter capsulatus* SB1003 was used as the wild-type parental strain with the Δ*aerR* and Δ*crtJ* single deletion strains and a Δ*aerR* Δ*crtJ* double deletion strain derived from SB1003 as described previously (20). *Escherichia coli* BL21(DE3) (15) was used to overexpress protein with *E. coli* S17-1 λpir (20) used to introduce different plasmids to *R. capsulatus* via conjugation. *R. capsulatus* strains were routinely grown in PY salts liquid medium at 34°C (15).

### Plasmid and strain construction.

Suicide plasmid pZJD29a was used to generate a nonpolar in-frame deletion and chromosomal tagged strain in *Rhodobacter capsulatus* SB1003 as previously reported (49). pSRKGm (50) was used to express 3×FLAG-tagged CrtJ in *R. capsulatus*, and pSUMO was used for overexpression of proteins in *E. coli* (15). The establishment of functional activity of the FLAG-tagged CrtJ and AerR constructs is provided in Text S2.

10.1128/mBio.00261-17.2 TEXT S2  Results of functional complementation of CrtJ and AerR deletion strains with 3×FLAG constructs of CrtJ and AerR, respectively. Download TEXT S2, PDF file, 1.4 MB. Copyright © 2017 Fang and Bauer. 2017 Fang and Bauer This content is distributed under the terms of the Creative Commons Attribution 4.0 International license.

### ChIP-seq and ChIP-exo.

Two epitope-tagged CrtJ constructs (N-terminal 3×FLAG and N-terminal 3×V5) were used in the experiments. In one set of experiments, FLAG-tagged CrtJ was expressed from the pSRKGm plasmid in three independent biological replicates by the addition of 100 μM IPTG added 30 min before formaldehyde cross-linking. In a second set of experiments, V5-tagged CrtJ was recombined into the chromosome at its native location from which it was expressed from its native promoter. Chromatin immunoprecipitation-DNA sequencing (ChIP-seq) analysis of AerR involved the use of a C-terminal 3×FLAG-tagged AerR construct expressed from pSRKGm with no IPTG induction. ChIP-seq and exonuclease digestion (ChIP-exo) procedures were performed using FLAG-tagged CrtJ as previously reported (35). Detailed procedures can be found in Text S1.

### RNA isolation and RNA-seq.

*Rhodobacter capsulatus* strains were grown to early exponential phase (optical density at 660 nm [OD_660_] of 0.3 to 0.35) before collection. Total RNA was extracted using Isolate II RNA minikit (Bioline) followed by Turbo DNase (Ambion) treatment. Library construction and transcriptome sequencing (RNA-seq) were performed by the University of Wisconsin Madison Biotechnology Center DNA Sequencing Facility using the TruSeq RNA sample preparation kit (Illumina) according to the manufacturer’s protocol.

### Protein purification, gel mobility shift assay, and DNase I footprint assay.

*E. coli* BL21(DE3) strains bearing plasmid pSUMO-AerR or pSUMO-CrtJ were used to overexpress SUMO-tagged AerR or CrtJ according to previous studies (15, 20). The gel mobility shift assay and DNase I footprint assay were performed as reported previously (20).

### Multiangle light scattering.

The size of AerR-CrtJ complex was determined by size exclusion chromatography coupled with multiangle light scattering (SEC-MALS) (Wyatt Technology Corp). The light scattering results were analyzed using ASTRA 6 (Wyatt Technology) with bovine serum albumin (BSA) for calibration.

### Accession number(s).

Raw sequence read files were deposited in the Sequence Read Archive (SRA) with the GenBank accession number SRP082587.
