# Supplementary material for: The Vitamin B12-Dependent Photoreceptor AerR Relieves Photosystem Gene Repression by Extending the Interaction of CrtJ with Photosystem Promoters
Source: mBio. 2017 Mar 21;8(2):e00261-17. doi: 10.1128/mBio.00261-17 (PMC5362033; doi:10.1128/mBio.00261-17)
Supplement: TABLE S1 [file mbo002173237st1.pdf]

Table S1. Differentially expressed genes in Δ*crtJ* strain under aerobic condition

Gene ID	Fold Change	p value	Annotation
RCAP_rcc00686	4.7	1.20E-19	*bchC*; 2-desacetyl-2-hydroxyethyl bacteriochlorophyllide A dehydrogenase
RCAP_rcc00695	4.0	2.48E-22	*pufX*; intrinsic membrane protein PufX
RCAP_rcc00694	3.9	5.19E-21	*pufM*; photosynthetic reaction center subunit M
RCAP_rcc00688	3.8	2.23E-24	*bchY*; chlorophyllide reductase subunit BchY (EC:1.18.1.-)
RCAP_rcc00693	3.8	8.20E-19	*pufL*; photosynthetic reaction center subunit L
RCAP_rcc00687	3.5	5.91E-15	*bchX*; chlorophyllide reductase subunit BchX (EC:1.3.1.-)
RCAP_rcc00696	3.5	7.10E-15	*dxs1*; 1-deoxy-D-xylulose-5-phosphate synthase (EC:2.2.1.7)
RCAP_rcc00692	3.5	1.45E-14	*pufA*; light-harvesting protein B-870 subunit alpha
RCAP_rcc00691	3.4	4.99E-14	*pufB*; light-harvesting protein B-870 subunit beta
RCAP_rcc00674	3.3	3.84E-22	*idi2*; isopentenyl-diphosphate delta-isomerase (EC:5.3.3.2)
RCAP_rcc00670	3.2	1.69E-17	*bchJ*; bacteriochlorophyll 4-vinyl reductase
RCAP_rcc00671	3.0	1.06E-13	*bchG*; bacteriochlorophyll synthase (EC:2.5.1.62)
RCAP_rcc00673	2.9	4.97E-16	*bchP*; geranylgeranyl reductase (EC:1.3.1.-)
RCAP_rcc00689	2.9	1.38E-08	*bchZ*; chlorophyllide reductase subunit BchZ (EC:1.18.-.-)
RCAP_rcc00672	2.8	1.77E-16	PUCC family protein chlorophyll transporter
RCAP_rcc02531	2.8	1.38E-14	*pucA*; light-harvesting protein B-800/850 subunit alpha
RCAP_rcc00665	2.7	1.91E-12	*bchN*; light-independent protochlorophyllide reductase N subunit (EC:1.3.1.33)
RCAP_rcc00690	2.7	3.34E-05	*pufQ*; cytochrome, subunit PufQ
RCAP_rcc00663	2.6	6.81E-12	*bchH*; magnesium chelatase H subunit (EC:6.6.1.1)
RCAP_rcc00661	2.6	1.78E-10	*bchM*; magnesium-protoporphyrin O-methyltransferase (EC:2.1.1.11)
RCAP_rcc00669	2.5	1.63E-05	*bchE*; magnesium-protoporphyrin IX monomethyl ester anaerobic oxidative cyclase (EC:1.14.13.81)
RCAP_rcc00662	2.5	3.31E-07	*bchL*; light-independent protochlorophyllide reductase iron-sulfur ATP-binding protein (EC:1.18.-.-)
RCAP_rcc00664	2.5	1.42E-12	*bchB*; light-independent protochlorophyllide reductase subunit B (EC:1.3.1.33)
RCAP_rcc00659	2.3	9.79E-08	*puhA*; photosynthetic reaction center subunit H
RCAP_rcc00666	2.3	0.000219	*bchF*; 2-vinyl bacteriochlorophyllide hydratase (EC:4.2.1.-)
RCAP_rcc03429	2.3	1.28E-13	hypothetical protein
RCAP_rcc00658	2.3	8.72E-07	hypothetical protein
RCAP_rcc00660	2.3	4.74E-06	*pucC1*; protein PucC chlorophyll transporter
RCAP_rcc02541	2.2	0.001115	*fruA*; PTS system fructose-specific transporter subunit EIIBC (EC:2.7.1.69)
RCAP_rcc00132	2.2	0.000917	hypothetical protein
RCAP_rcc00676	2.1	4.32E-05	*bchD*; magnesium chelatase ATPase subunit D (EC:6.6.1.1)
RCAP_rcc02530	2.1	0.000262	*pucB*; light-harvesting protein B-800/850 subunit beta

RCAP_rcc02543	2.1	0.002852	*fruB*; multiphosphoryl transfer protein (EC:2.7.1.- 2.7.3.9)
RCAP_rcc00677	2.0	1.94E-06	*bchI*; magnesium chelatase ATPase subunit I (EC:6.6.1.1)
RCAP_rcc02415	2.0	0.009707	hypothetical protein
RCAP_rcc02532	2.0	0.001152	*pucC2*; protein PucC chlorophyll transporter
RCAP_rcc00684	2.0	0.000843	*crtE*; farnesyltranstransferase (EC:2.5.1.29)
RCAP_rcc00655	2.0	2.38E-05	hypothetical protein
RCAP_rcc00679	2.0	2.64E-08	*crtI*; phytoene dehydrogenase (EC:1.14.99.-)
RCAP_rcc00656	1.9	4.21E-06	hypothetical protein
RCAP_rcc02533	1.9	0.000175	*pucDE*; light-harvesting protein B-800/850 subunit gamma
RCAP_rcc00678	1.9	0.006005	*crtA*; spheroidene monooxygenase
RCAP_rcc00680	1.9	0.008258	*crtB*; phytoene synthase (EC:2.5.1.32)
RCAP_rcc00685	1.8	4.21E-06	*crtF*; hydroxyneurosporene methyltransferase (EC:2.1.1.-)
RCAP_rcc00683	1.8	3.45E-05	*crtD*; methoxyneurosporene dehydrogenase (EC:1.14.99.-)
RCAP_rcc00657	1.8	0.000917	hypothetical protein
RCAP_rcc00682	1.7	0.001683	*crtC*; hydroxyneurosporene synthase
RCAP_rcc02015	1.7	0.000409	*aldH1*; aldehyde dehydrogenase (EC:1.2.1.3)
RCAP_rcc01160	-1.7	0.000222	*ccoP; cbb3*-type cytochrome c oxidase subunit III (EC:1.9.3.1)
RCAP_rcc01157	-1.8	0.000262	*ccoN; cbb3*-type cytochrome c oxidase subunit I (EC:1.9.3.1)
RCAP_rcc00103	-1.8	0.000419	ABC transporter periplasmic substrate-binding protein
RCAP_rcc01159	-1.8	0.002722	*ccoQ; cbb3*-type cytochrome c oxidase subunit IV (EC:1.9.3.1)
RCAP_rcc01158	-1.9	1.94E-06	*ccoO*; *cbb3*-type cytochrome c oxidase subunit II (EC:1.9.3.1)
RCAP_rcc00099	-1.9	0.001143	ABC transporter ATP-binding protein (EC:3.6.3.-)
