# Supplementary material for: The Vitamin B12-Dependent Photoreceptor AerR Relieves Photosystem Gene Repression by Extending the Interaction of CrtJ with Photosystem Promoters
Source: mBio. 2017 Mar 21;8(2):e00261-17. doi: 10.1128/mBio.00261-17 (PMC5362033; doi:10.1128/mBio.00261-17)
Supplement: TABLE S2 [file mbo002173237st2.pdf]

Table S2. Differentially expressed genes in Δ*crtJ* strain under anaerobic photosynthetic condition

Gene ID	Fold Change	p value	Annotation
RCAP_rcc01445	2.6	3.00E-06	TonB-dependent receptor
RCAP_rcc02565	2.5	0.002131	hypothetical protein
RCAP_rcc02293	2.4	0.001180	hypothetical protein
RCAP_rcc02285	2.4	0.003380	hypothetical protein
RCAP_rcc03387	2.4	6.13E-09	*glnB2*; nitrogen regulatory protein P-II
RCAP_rcc03248	2.3	0.002729	metallophosphoesterase
RCAP_rcc00737	2.2	9.31E-06	hypothetical protein
RCAP_rcc01317	2.1	0.004170	pirin domain-containing protein
RCAP_rcc00353	2.1	1.62E-08	hypothetical protein
RCAP_rcc02413	2.1	3.34E-05	hypothetical protein
RCAP_rcc02154	2.1	9.29E-09	TM2 domain-containing protein
RCAP_rcc02385	2.0	0.000127	hypothetical protein
RCAP_rcc02923	2.0	1.34E-11	hypothetical protein
RCAP_rcc00981	2.0	1.11E-05	phage head morphogenesis protein
RCAP_rcc02288	2.0	2.58E-08	hypothetical protein
RCAP_rcc02808	2.0	1.78E-10	hypothetical protein
RCAP_rcc00234	1.9	0.008509	luciferase
RCAP_rcc01184	1.9	2.89E-06	CsbD family protein
RCAP_rcc02567	1.9	3.69E-07	resolvase
RCAP_rcc00263	1.9	1.67E-12	winged helix family two component transcriptional regulator
RCAP_rcc01310	1.8	0.004857	hypothetical protein
RCAP_rcc03212	1.8	0.001180	*prmA*; ribosomal protein L11 methyltransferase (EC:2.1.1.-)
RCAP_rcc00984	1.8	0.007822	hypothetical protein

RCAP_rcc01666	1.8	5.05E-05	hypothetical protein
RCAP_rcc01012	1.8	8.25E-05	hypothetical protein
RCAP_rcc01301	1.8	0.005378	Cas5 family CRISPR-associated protein
RCAP_rcc00080	1.8	0.000508	hypothetical protein
RCAP_rcc01181	1.8	5.24E-06	hypothetical protein
RCAP_rcc03259	1.8	0.001705	*msrB1*; peptide-methionine (R)-S-oxide reductase (EC:1.8.4.12)
RCAP_rcc02566	1.7	1.24E-07	reverse transcriptase catalytic domain-containing protein (EC:2.7.7.49)
RCAP_rcc00604	1.7	2.83E-08	TerC family integral membrane protein
RCAP_rcc01038	1.7	0.001200	hypothetical protein
RCAP_rcc00572	1.7	0.007851	*nifH1*; nitrogenase iron protein (EC:1.18.6.1)
RCAP_rcc01220	1.7	1.17E-05	*ureC*; urease subunit alpha (EC:3.5.1.5)
RCAP_rcc02626	1.7	0.000996	hypothetical protein
RCAP_rcc01347	1.7	0.003380	pirin domain-containing protein; K06911
RCAP_rcc00280	1.7	9.29E-09	hemolysin-type calcium-binding repeat family protein
RCAP_rcc03352	1.7	0.003208	*metH2*; methionine synthase subunit B (EC:2.1.1.13)
RCAP_rcc03055	1.7	1.84E-05	*dnaG*; DNA primase (EC:2.7.7.-)
RCAP_rcc00686	1.7	0.008707	*bchC*; 2-desacetyl-2-hydroxyethyl bacteriochlorophyllide A dehydrogenase
RCAP_rcc02275	1.7	5.79E-05	*oppA2*; oligopeptide ABC transporter periplasmic oligopeptide-binding protein OppA
RCAP_rcc03327	1.7	1.52E-07	*lolA*; outer membrane lipoprotein carrier protein LolA
RCAP_rcc02495	1.6	0.000864	GTPase, EngC family (EC:3.6.1.-)
RCAP_rcc02811	1.6	1.31E-06	*rpoH2*; RNA polymerase sigma-32 factor
RCAP_rcc03024	1.6	0.006407	*dctP3*; TRAP C4-dicarboxylate transport system permease subunit DctP
RCAP_rcc02702	1.6	0.001200	cytochrome c/b561 family protein
RCAP_rcc01622	1.6	0.007650	hypothetical protein
RCAP_rcc03326	1.6	0.009944	lipoprotein
RCAP_rcc03386	1.6	0.000127	*amtB*; ammonium transporter
RCAP_rcc01930	1.5	0.000963	*hsdM3*; type I restriction-modification system RcaSBIV subunit M (EC:2.1.1.72)

RCAP_rcc01881	1.5	5.87E-05	*sufB*; FeS assembly protein SufB
RCAP_rcc00748	1.5	0.004981	*phaR*; polyhydroxyalkanoate synthesis repressor PhaR
RCAP_rcc02388	1.5	0.003391	*ispZ*; intracellular septation protein A
RCAP_rcc03385	1.5	0.002150	*emrB*; multidrug resistance protein B (EC:3.6.3.44)
RCAP_rcc02818	1.5	0.000734	*ibpA*; small heat shock protein IbpA
RCAP_rcc00357	1.5	0.006336	*macB*; macrolide export ABC transporter ATP-binding/permease MacB (EC:3.6.3.-)
RCAP_rcc03380	1.5	6.30E-06	*smpB*; ssrA-binding protein
RCAP_rcc00602	1.5	0.003242	*mntR*; transcriptional regulator MntR
RCAP_rcc00384	1.5	0.000203	*gshA*; glutamate--cysteine ligase (EC:6.3.2.2)
RCAP_rcc03141	1.5	0.009542	*cspD*; cold shock-like protein CspD
RCAP_rcc01800	1.5	0.000161	*ntrX*; nitrogen assimilation regulatory protein NtrX
RCAP_rcc03304	1.4	0.006853	hypothetical protein
RCAP_rcc01700	1.4	0.001200	*cysE1*; serine O-acetyltransferase (EC:2.3.1.30)
RCAP_rcc00745	1.4	0.007414	*phaZ*; polyhydroxyalkanoate depolymerase (EC:3.1.1.75)
RCAP_rcc03390	1.4	0.008707	VacJ family lipoprotein
RCAP_rcc00687	1.4	0.007825	*bchX*; chlorophyllide reductase subunit BchX (EC:1.3.1.-)
RCAP_rcc00026	-1.5	0.008707	hypothetical protein
RCAP_rcc02887	-1.5	0.002843	methyl-accepting chemotaxis sensory transducer
RCAP_rcc02065	-1.5	0.000914	secretion ATP-binding protein, HlyB family (EC:3.6.3.-)
RCAP_rcc02611	-1.5	0.001842	*mcpA3*; methyl-accepting chemotaxis protein McpA
RCAP_rcc02857	-1.6	0.000538	diguanylate cyclase/phosphodiesterase (EC:3.1.4.-)
RCAP_rcc03528	-1.6	2.83E-05	hypothetical protein
RCAP_rcc01667	-1.7	0.002318	methyl-accepting chemotaxis sensory transducer
RCAP_rcc02063	-1.7	0.000963	M10 family peptidase (EC:3.4.24.-)
RCAP_rcc00620	-1.7	0.001796	response regulator receiver modulated diguanylate cyclase/phosphodiesterase (EC:3.1.4.-)
RCAP_rcc02435	-1.7	0.001200	family 2 glycosyl transferase
RCAP_rcc02069	-1.7	0.002678	hypothetical protein

RCAP_rcc00645	-1.7	0.000638	diguanylate cyclase/phosphodiesterase (EC:3.1.4.-)
RCAP_rcc02596	-1.7	0.009393	lipoprotein
RCAP_rcc01356	-1.8	0.003765	chemotaxis protein CheW
RCAP_rcc00244	-1.8	0.007671	hypothetical protein
RCAP_rcc00564	-1.8	0.006336	*modC1*; molybdenum ABC transporter ATP-binding protein ModC (EC:3.6.3.29)
RCAP_rcc00844	-1.8	0.008782	hypothetical protein
RCAP_rcc02068	-1.9	3.31E-05	ice nucleation protein repeat family protein
RCAP_rcc01382	-2.0	0.007241	hypothetical protein
RCAP_rcc01033	-2.1	0.001796	*cbiQ1*; cobalt ABC transporter permease CbiQ
RCAP_rcc03514	-2.1	0.003475	*flgA*; flagella basal body P-ring formation protein FlgA
RCAP_rcc02211	-2.2	2.39E-05	*pflD*; formate C-acetyltransferase (EC:2.3.1.54)
RCAP_rcc02208	-3.1	1.20E-05	*pduB*; propanediol utilization protein PduB
