# Supplementary material for: The Vitamin B12-Dependent Photoreceptor AerR Relieves Photosystem Gene Repression by Extending the Interaction of CrtJ with Photosystem Promoters
Source: mBio. 2017 Mar 21;8(2):e00261-17. doi: 10.1128/mBio.00261-17 (PMC5362033; doi:10.1128/mBio.00261-17)
Supplement: TABLE S3 [file mbo002173237st3.pdf]

Table S3. Called CrtJ ChIP-Seq peaks under aerobic conditions.

Peak summit position	Fold enrichment	CrtJ binding sequence	AerR colocalization	Gene annotation
200	7.36	TGTGATCCGTTTCAAATG	-	*dnaA*; chromosomal replication initiator protein DnaA
55617	4.53	TGGAGGTTTTCATGGATA	-	*hvrA*; trans-acting regulatory protein HvrA
73393	3.39	TGTTTCACGTGAAACACT	-	*gidA*; tRNA uridine 5-carboxymethylaminomethyl modification enzyme GidA
123748	2.89	GGTTGCGGGACAATGACG	-	118; sigma 54 modulation protein/ribosomal protein S30EA
222690	10.05	TGTATTTCGAATACCTCA	-	185; hypothetical protein
245576	4.23	CGTTTCGGGGTCCAAACC	-	212; hypothetical protein
296520	3.16	TGTACGACAGCGAAGACT	-	259; porin family protein
318114	3.46	TGTCGATCACCGTCGAGA	-	*rpsO*; 30S ribosomal protein S15
323398	5.5		+	*nusG*; transcription antitermination protein NusG
336284	3.17	TGTGCACGCGCGTGTACA	-	*rplL*; 50S ribosomal protein L7/L12
346525	3.39	TGTCTTTCTGGTTCGTCA	-	*rplO*; 50S ribosomal protein L15
354541	3.59	TGGAAGCACGGAACCGCA	-	*rpsM*; 30S ribosomal protein S13
422245	4.48	TGTAAACAAAGGGTTACA	-	*uvrC*; UvrABC system protein C
496480	2.95	TGGTCAGTTCGCGGCACA	-	*gshB*; glutathione synthase (EC:6.3.2.3)
513708	4.71	TGAAGAAAGCGCGCGAGA	-	479; COQ9 family ubiquinone biosynthesis protein
517964	6.8	TCTCGCTCAATCCCAACA	-	*gst*; glutathione S-transferase (EC:2.5.1.18)
526234	6.33	GGTGGGGAGACGCTTACG	+	*ldc*; lysine/ornithine decarboxylase (EC:4.1.1.17 4.1.1.18)
596988	3.95	AGTTCTGATCCCTGAACC	+	*metG*; methionyl-tRNA synthetase (EC:6.1.1.10)
644776	5.78	CGTCTGCCGGGCCGCACC	-	*nrdJ1*; ribonucleoside-diphosphate reductase NrdJ (EC:1.17.4.1)
649580	4.57	TGTCGATGCGGTCCGACA	-	*kup*; potassium transporter
652660	6.36	AGTTTTTCAACCAGAACA	-	*cspA1*; cold shock protein CspA
702161	6.57	TTTTACAAAAGATATACA	-	642; peptidoglycan binding domain-containing protein
735134	3.43	TGTCAATGAAAACTTACA	-	*bchF*; 2-vinyl bacteriochlorophyllide hydratase (EC:4.2.1.-)
738276	6.43	TGTCAACTGAGGTTTACA	-	*bchE*; magnesium-protoporphyrin IX monomethyl ester anaerobic oxidative cyclase

749266	3.99	TGTAACGGGATATTTACA	-	*crtA*; spheroidene monooxygenase
755161	5.31	TGTAAGTTTCAGTTTACA	-	*crtD*; methoxyneurosporene dehydrogenase (EC:1.14.99.-)
757360	11.04	TGTCTAATCAAATTGACA	-	*bchC*; 2-desacetyl-2-hydroxyethyl bacteriochlorophyllide A dehydrogenase;
810627	4.4	TGGATGCCGTCTTTGGCA	+	*atpB*; ATP synthase F0 subunit A (EC:3.6.3.14)
820770	4.53	GGTGATGAAGACATAACG	-	*pyrG*; CTP synthase (EC:6.3.4.2)
825549	5.36	GGTGCTTTCCGGTGAACG	-	*thiN*; thiamine pyrophosphokinase (EC:2.7.6.2)
833335	6.74	TGTCAATTTTCTCTTACA	-	*mcpA1*; methyl-accepting chemotaxis protein McpA
906176	6.37	GGTACTCATGGGCGGACC	+	*ddl*; D-alanine--D-alanine ligase (EC:6.3.2.4)
909431	4.85	TGTGTTTTTTGGGTGACG	-	*ftsZ*; cell division protein FtsZ
932735	2.9	TGTGTGAGTAGAAATAAA	-	842; hypothetical protein
958344	3.66	TGGAAAGCGGATCGGACT	-	*adhC*; alcohol dehydrogenase/S-(hydroxymethyl)glutathione dehydrogenase
961088	2.92	TGCCGAAGATCGCGGCCA	-	871; hypothetical protein
962568	2.51	GGTGGGGGTGACGCAACA	-	873; XRE family transcriptional regulator
993044	5.05		-	*pccB*; propionyl-CoA carboxylase subunit beta (EC:6.4.1.3)
1025688	2.84	TGTTGCCTTATTGTAACG	-	930; hypothetical protein
1043248	2.61	TTTCTGAAGACCCGAACA	-	*hsdR1*; type I restriction-modification system RcaSBIP subunit R (EC:3.1.21.3)
1091014	4.57		-	1010; DNA binding protein
1151106	6.6	TGGTTCTGGTCTGCAACA	-	1081; group 1 glycosyl transferase (EC:2.4.1.-)
1189678	3		-	*corC*; magnesium and cobalt efflux protein CorC
1212863	3.48	CGTCAGGATCGGGAGACG	-	*gcvT1*; glycine cleavage T protein (EC:2.1.2.10)
1226734	5.36	TGCCGTATATGGTGAACA	-	*ccoN*; cbb3-type cytochrome c oxidase subunit I (EC:1.9.3.1)
1230352	3.17	CGTCGGTGCGTTTCAACA	-	*ccoG*; cbb3-type cytochrome c oxidase accessory protein CcoG
1236434	6.19	TGTATCCGACGTTTGCCA	-	*dacC1*; D-alanyl-D-alanine carboxypeptidase (EC:3.4.16.4)
1242827	8.64	TGTCAAGCACTGTTGACA	-	*hemE*; uroporphyrinogen decarboxylase (EC:4.1.1.37)
1285342	7.81	CGTTTTTCCTGCGTTACC	+	1209; cell wall hydrolase, SleB
1305978	4.78	GGTCGCGCCGACGCCACA	-	1234; hypothetical protein
1315973	8		-	*potA1*; polyamine ABC transporter ATP binding protein PotA (EC:3.6.3.31)

1346374	5.01	GGTGTTCCGCGCATGACC	-	1268; hypothetical protein
1349870	10.17	GGTGACGCATCCTTGACG	+	1272; hypothetical protein
1358507	3.09	AGTAAGCGGATTGAGACC	-	1277; RNA-directed DNA polymerase (EC:2.7.7.49)
1401501	3.73	AGTCCCTTCCTACTTACT	-	1311; hypothetical protein
1431402	3.21	GGTGTTCACGGATTTACT	-	1330; hypothetical protein
1565016	6.97	CGTCAAGAATTCGGGACC	-	1448; hypothetical protein
1573688	10.45	GGTCGATTCGGCCGGACA	+	1452; acriflavin resistance protein family
1584750	4.12	AGTTTCAGCGTGATGACG	-	1463; hypothetical protein
1644658	4.44	TGTCGATCCTGTTCATCA	+	*nuoA*; NADH-quinone oxidoreductase subunit A (EC:1.6.99.5)
1748859	5.98	AGTTTCAGGTCGTGGACA	-	*gppA*; guanosine-5'-triphosphate,3'-diphosphate pyrophosphatase (EC:3.6.1.40)
1800939	2.99	TGTTGGTGGAGGACGACC	-	*ctrA*; cell cycle transcriptional regulator CtrA
1815243	4.08	TGGGCGTGGAAGAGGACA	-	*acpP1*; acyl carrier protein
1818586	4.65	TGCTAGGAGAGGTGGGCA	-	1682; hypothetical protein
1915138	6.57		-	*aglE*; alpha-glucoside ABC transporter substrate-binding protein
1964315	6.43	TGTATGAAACCTGCCTCA	-	*bdhA*; 3-hydroxybutyrate dehydrogenase (EC:1.1.1.30)
1971058	3.36	TGTCGCCCCCTTGGCGCA	-	1823; LuxR family autoinducer-binding transcriptional regulator
1997996	6	GGTTTCCGTCGCAGTACC	-	1845; hypothetical protein
2027936	9.99	GGTCCGGTTTTCCCTACC	+	1874; hypothetical protein
2035474	2.72	TGTCCTGTTCCTGCGTCA	-	*sufB*; FeS assembly protein SufB
2057394	7.13	GGTCACGGCAGCACCACT	-	1900; hemolysin-type calcium-binding repeat family protein (EC:4.6.1.1)
2064384	3.66	GGTCAAACTGTTCAAACA	-	*rpmF*; 50S ribosomal protein L32
2067094	5.35	CGTTTTCCGTGCGCGACA	-	*ihfA*; integration host factor subunit alpha
2075449	4.27	TGATGGCGGCATGCAACA	-	1919; membrane protein involved in aromatic hydrocarbon degradation
2157906	3.87	TGTGCTGGGGGCGCGACA	-	2005; hypothetical protein
2162584	5.33	TGAACCTGCCGGGTCTCA	-	2006; hypothetical protein
2169146	5.52	TGACGCGCAATGAGGACA	-	*rpsF*; 30S ribosomal protein S6
2197210	3.18	CGTCACGCCGCGGGCACA	-	2037; CbiM family cobalamin biosynthesis protein

2279645	4.75	CGTGATTCCCCTTTGACC	-	2119; type 12 family methyltransferase (EC:2.1.1.-)
2337323	6.12	TGTCCTCCTCTCAGGACG	-	*rne*; ribonuclease E (EC:3.1.4.-)
2474681	4.8	GGTTTCCCGCCTCGGACA	-	*rplU*; 50S ribosomal protein L21
2555238	5.85	TGTATTCGCCGATGAACA	-	*mraZ*; protein MraZ
2676127	3.43	TCTCGCATCTTGCGCAGA	-	*hemN2*; oxygen-independent coproporphyrinogen-III oxidase (EC:1.3.99.22)
2677815	4.81	TGTCCTGCGAGGATCACC	-	2495; GTPase, EngC family (EC:3.6.1.-)
2720303	3.46	TGTACATCCCGCATGACA	-	*pucB*; light-harvesting protein B-800/850 subunit beta
2755117	4.21	TGTGCTGAGGGCTTCACG	-	2566; reverse transcriptase catalytic domain-containing protein (EC:2.7.7.49)
2796328	3.52		-	*clpX*; ATP-dependent Clp protease ATP-binding subunit ClpX (EC:3.4.21.92)
2825249	4.78	GGTTAAAGGAGCGGAACG	-	2634; response regulator receiver modulated diguanylate cyclase/phosphodiesterase
2863519	4.21		-	2670; Fur family transcriptional regulator
2939422	7.44	GGTCCGCAAGATCTTACT	-	*rpmG*; 50S ribosomal protein L33
2981918	8.25	GGTTACGGCCCCATCACA	+	2790; CarD family transcriptional regulator
3090712	5.28	TGCCCGCAGAATGCGACA	-	*cspA3*; cold shock protein CspA
3130838	3.15	GGTAGTTACCAAAGAACA	-	2938; hypothetical protein
3163985	3.39	GGTTCGGACACGTCCACC	+	*atpH*; ATP synthase F1 subunit delta (EC:3.6.3.14)
3266463	9.1	TGCCGCAGATGCGCGACA	+	3072; hypothetical protein
3308066	5.49	TGCCGGACATGCCTTTCA	-	gcvT2; glycine cleavage T protein (EC:2.1.2.10)
3310279	5.23	TGCTCTCTGGCCGACACA	-	3113; hypothetical protein
3321654	4.24		-	3125; heavy metal transport/detoxification protein family
3323346	5.36	TGTCGGGCAACAACATCA	+	*rpmB*; 50S ribosomal protein L28
3360695	5.5	TGTCGGCCGTTACGGAAA	+	3162; mandelate racemase/muconate lactonizing enzyme family protein
3379732	4.74	TGTTTCTTGCGAAGTTCA	-	3187; AlgR/AgrA/LytR family transcriptional regulator
3499282	4.84	TGTTGCCCCCGGGTCACC	-	*dapE*; succinyl-diaminopimelate desuccinylase (EC:3.5.1.18)
3511344	3	GGTCGATCTCGCCCGACA	-	*spoT*; bifunctional alarmone synthase/hydrolase (EC:2.7.6.5)
3548757	6.51		-	metH1; methionine synthase subunit A (EC:2.1.1.13)
3631764	6.36	TGTAAACTAAAATGGACA	+	livM3; branched-chain amino acid ABC transporter permease LivM

3644556	4.22	AGTTGGATTTTCCGGACA	-	3441; hypothetical protein
3725486	2.95		-	*flgB*; flagellar basal-body rod protein; FlgB
3728469	2.75		-	*flaA*; flagellin protein
