# Supplementary material for: The Vitamin B12-Dependent Photoreceptor AerR Relieves Photosystem Gene Repression by Extending the Interaction of CrtJ with Photosystem Promoters
Source: mBio. 2017 Mar 21;8(2):e00261-17. doi: 10.1128/mBio.00261-17 (PMC5362033; doi:10.1128/mBio.00261-17)
Supplement: TABLE S4 [file mbo002173237st4.pdf]

Table S4. CrtJ ChIP-Seq peaks under photosynthetic condition.

Peak summit position	Fold enrichment	CrtJ binding sequence	AerR colocalization	Gene annotation
199	4.4	TGTGATCCGTTTCAAATG	−	*dnaA*; chromosomal replication initiator protein DnaA
22293	3.42	TGTCTAAGCGGCCCTACG	+	*xylF*; xylose ABC transporter xylose-binding protein XylF
56948	4.94		+	*hvrB*; AHCY transcriptional activator HvrB
73386	4.06	TGTTTCACGTGAAACACT	−	*gidA*; tRNA uridine 5-carboxymethylaminomethyl modification enzyme GidA
88605	4.63	CGTCGTCGAGGGAAGACA	−	79; iojap-related protein
123717	3.72	GGTTGCGGGACAATGACG	−	118; sigma 54 modulation protein/ribosomal protein S30EA
222675	7.93	TGTATTTCGAATACCTCA	+	185; hypothetical protein
245582	4.22	CGTTTCGGGGTCCAAACC	−	212; hypothetical protein
262187	2.37	TGTCCACCCCCGCCCAGG	−	*dnaK*; chaperone DnaK
296580	5.23	TGTACGACAGCGAAGACT	+	259; porin family protein
317628	2.93	TGTCTTTCTGGTTCGTCA	−	*rpsO*; 30S ribosomal protein S15
326580	2.38	TGTGAGTTCTTGGCCGCA	+	*nusG*; transcription antitermination protein NusG
336255	4.86	AGTATTGACAGCAGAACG	−	*rpsL*; 30S ribosomal protein S12
377470	3.02		+	*rpmH*; 50S ribosomal protein L34
402074	3.52	TGGGCAGCCAGTTCGACA	+	*mdoH*; glucans biosynthesis glucosyltransferase H (EC:2.4.1.-)
422221	5.09	TGTAAACAAAGGGTTACA	+	*uvrC*; UvrABC system protein C
444721	7.31	TGCCATCGAAATTTGTCA	−	416; LysR family transcriptional regulator
454760	5.84		+	424; hypothetical protein
465179	3.6	TGAGCAGACGTTTGATCA	−	434; hypothetical protein
496485	4.32	TGGTCAGTTCGCGGCACA	+	*gshB*; glutathione synthase (EC:6.3.2.3)
526276	3.95	GGTGGGGAGACGCTTACG	+	*ldc*; lysine/ornithine decarboxylase (EC:4.1.1.17 4.1.1.18)
581207	3.55	TGTGAGTTTTCCTGACCA	−	542; hypothetical protein
586699	3.32	TGGTTGCCGTCCCCGGCA	−	548; BolA family protein
589316	5		−	*purL*; phosphoribosylformylglycinamidine synthase II (EC:6.3.5.3)

596974	5.7	AGTTCTGATCCCTGAACC	−	*metG*; methionyl-tRNA synthetase (EC:6.1.1.10)
644791	3.16	CGTCTGCCGGGCCGCACC	−	*nrdJ1*; ribonucleoside-diphosphate reductase NrdJ (EC:1.17.4.1)
649595	4.26	TGTCGATGCGGTCCGACA	+	*kup*; potassium transporter
652570	4.69	AGTTTTTCAACCAGAACA	−	*cspA1*; cold shock protein CspA
725096	3.3		−	*puhA*; photosynthetic reaction center subunit H
735139	4.43	TGTCAATGAAAACTTACA	+	*bchF*; 2-vinyl bacteriochlorophyllide hydratase (EC:4.2.1.-)
738262	10.98	TGTCAACTGAGGTTTACA	+	*bchE*; magnesium-protoporphyrin IX monomethyl ester anaerobic oxidative cyclase
749193	5.71	TGTAACGGGATATTTACA	+	*crtA*; spheroidene monooxygenase
755202	8.34	TGTAAGTTTCAGTTTACA	+	*crtD*; methoxyneurosporene dehydrogenase (EC:1.14.99.-)
757374	9.51	TGTCTAATCAAATTGACA	+	*bchC*; 2-desacetyl-2-hydroxyethyl bacteriochlorophyllide A dehydrogenase
762248	4.99		+	*pufQ*; cytochrome, subunit PufQ
781576	3.94	TGGCGCAATTCGGCATCA	−	711; universal stress family protein
810634	3.4	TGGATGCCGTCTTTGGCA	−	*atpB*; ATP synthase F0 subunit A (EC:3.6.3.14)
814518	4.64	TGTTCCTGATGGTTTCCA	−	*phbC*; poly(3-hydroxyalkanoate) polymerase (EC:2.3.1.-)
825470	4.37	GGTGCTTTCCGGTGAACG	−	*thiN*; thiamine pyrophosphokinase (EC:2.7.6.2)
834185	4.76	TGTCAATTTTCTCTTACA	−	*mcpA1*; methyl-accepting chemotaxis protein McpA
842610	4.53	TGACAAAATCACACACCA	−	*hupA*; hydrogenase small subunit (EC:1.12.99.6)
846509	6.27		−	*hupD*; hydrogenase maturation protease HupD (EC:3.4.23.-)
906046	4.59	GGTACTCATGGGCGGACC	−	*ddl*; D-alanine--D-alanine ligase (EC:6.3.2.4)
909454	4.8	TGTGTTTTTTGGGTGACG	−	*ftsZ*; cell division protein FtsZ
911425	4.05	GGTCAACGGTTCTTAACT	−	*lpxC*; UDP-3-O-[3-hydroxymyristoyl] N-acetylglucosamine deacetylase (EC:3.5.1.-)
960910	3.2	TGCCGAAGATCGCGGCCA	−	871; hypothetical protein
988886	8.44		+	901; hypothetical protein
993069	4.86	TGACGAGCGGCGCAGGCA	−	*pccB*; propionyl-CoA carboxylase subunit beta (EC:6.4.1.3)
995417	3.88	GGTCTTCCAAGCGGAACT	−	910; hypothetical protein
1072710	4.56		+	980; phage virion morphogenesis protein
1151113	6.12		+	1081; group 1 glycosyl transferase (EC:2.4.1.-)

1226508	6.41	TGCCGTATATGGTGAACA	+	*ccoN*; cbb3-type cytochrome c oxidase subunit I (EC:1.9.3.1)
1236458	5.65	TGTATCCGACGTTTGCCA	+	*dacC1*; D-alanyl-D-alanine carboxypeptidase (EC:3.4.16.4)
1242843	6.66	TGTCAAGCACTGTTGACA	+	*hemE*; uroporphyrinogen decarboxylase (EC:4.1.1.37)
1285344	4.62	CGTTTTTCCTGCGTTACC	+	1209; cell wall hydrolase, SleB
1315971	5.03	TGTGCAGAAAAGCTATGA	−	*potA1*; polyamine ABC transporter ATP binding protein PotA (EC:3.6.3.31)
1346401	4.98	GGTGTTCCGCGCATGACC	−	1268; hypothetical protein
1349869	4.97	GGTGACGCATCCTTGACG	+	1272; hypothetical protein
1356497	3.81	TGCGATCAGGCCCAGACA	−	1276; Cas1 family CRISPR-associated protein
1358542	3.86	AGTAAGCGGATTGAGACC	−	1277; RNA-directed DNA polymerase (EC:2.7.7.49)
1401438	2.9	AGTCCCTTCCTACTTACT	+	1311; hypothetical protein
1565022	6.16	CGTCAAGAATTCGGGACC	+	1448; hypothetical protein
1573615	8.33	GGTCGATTCGGCCGGACA	+	1452; acriflavin resistance protein family
1589373	9.28		−	*gcvA*; glycine cleavage system transcriptional activator
1619988	3.69	TCTCGAAGGCCTGCAACA	−	*fusA2*; translation elongation factor G (EC:3.6.5.3)
1644564	2.62	TGTCGATCCTGTTCATCA	−	*nuoA*; NADH-quinone oxidoreductase subunit A (EC:1.6.99.5)
1710575	5.38	TGTTCGAAGCGCCCGCCA	+	1579; hypothetical protein
1748855	5.95	AGTTTCAGGTCGTGGACA	+	*gppA*; guanosine-5'-triphosphate,3'-diphosphate pyrophosphatase (EC:3.6.1.40)
1800758	2.74	TGTTGGTGGAGGACGACC	−	*ctrA*; cell cycle transcriptional regulator CtrA
1811125	3.13	TGGTGATACGAGGGCGCA	−	*glnB1*; nitrogen regulatory protein P-II
1815236	4.41	TGGGCGTGGAAGAGGACA	−	*acpP1*; acyl carrier protein
1818597	4.53	TGCTAGGAGAGGTGGGCA	−	1682; hypothetical protein
1885733	6.29	TGCAAAAAGATGTTAACA	−	1746; hypothetical protein
1915098	5.89		−	*aglE*; alpha-glucoside ABC transporter substrate-binding protein
1971045	3.51	TGTCGCCCCCTTGGCGCA	−	1823; LuxR family autoinducer-binding transcriptional regulator
1998003	4.87	GGTTTCCGTCGCAGTACC	−	1845; hypothetical protein
2027920	6.35	GGTCCGGTTTTCCCTACC	+	1874; hypothetical protein
2057437	3.71	GGTCACGGCAGCACCACT	−	1900; hemolysin-type calcium-binding repeat family protein (EC:4.6.1.1)

2064116	3.6	GGTCAAACTGTTCAAACA	−	*rpmF*; 50S ribosomal protein L32
2067079	3.53	CGTTTTCCGTGCGCGACA	+	*ihfA*; integration host factor subunit alpha
2075461	3.81	TGATGGCGGCATGCAACA	−	1919; membrane protein involved in aromatic hydrocarbon degradation
2157897	3.84	TGTGCTGGGGGCGCGACA	−	2005; hypothetical protein
2162537	4.88	TGAACCTGCCGGGTCTCA	+	2006; hypothetical protein
2197216	5.11	CGTCACGCCGCGGGCACA	−	2037; CbiM family cobalamin biosynthesis protein
2243758	4.25	TGTCGGTGCGCGAAAAGA	−	2075; PAS/PAC sensor domain-containing protein
2279721	4.04	CGTGATTCCCCTTTGACC	−	2119; type 12 family methyltransferase (EC:2.1.1.-)
2322255	4.04	TGCGCTTTACATCGAACA	−	2154; TM2 domain-containing protein
2329914	4.11	GGTGCGGCGCAGACAACC	−	2161; hypothetical protein
2337317	5.25	TGTCCTCCTCTCAGGACG	+	*rne*; ribonuclease E (EC:3.1.4.-)
2474661	4.35	GGTTTCCCGCCTCGGACA	+	*rplU*; 50S ribosomal protein L21
2495432	4.48		+	*prfC*; peptide chain release factor 3
2499201	2.96	TGTTGGGAAGGTGAACCA	−	2326; phage integrase
2555242	5.14	TGTATTCGCCGATGAACA	+	*mraZ*; protein MraZ
2591042	2.41	TGCGCGAAGCCCGCGACA	−	2415; hypothetical protein
2637738	3.87	TATTGGCCGAAGGCGACA	+	*cspA2*; cold shock protein CspA
2676231	4.12	TCTCGCATCTTGCGCAGA	−	*hemN2*; oxygen-independent coproporphyrinogen-III oxidase (EC:1.3.99.22)
2699900	3.24	TGTGCGCCATGAAAGACG	−	*rhlE*; ATP-dependent RNA helicase RhlE (EC:3.6.1.-)
2720366	7.85	TGTACATCCCGCATGACA	+	*pucB*; light-harvesting protein B-800/850 subunit beta
2755114	6.57	TGTGCTGAGGGCTTCACG	+	2566; reverse transcriptase catalytic domain-containing protein (EC:2.7.7.49)
2780075	9.85	TGTCCTGCCCGATCGAAA	+	*dksA2*; DnaK suppressor protein
2796395	3.32		−	*clpX*; ATP-dependent Clp protease ATP-binding subunit ClpX (EC:3.4.21.92)
2825212	6.31	GGTTAAAGGAGCGGAACG	−	2634; response regulator receiver modulated diguanylate cyclase/phosphodiesterase
2939495	3.92	GGTCCGCAAGATCTTACT	+	*rpmG*; 50S ribosomal protein L33
2952981	10.32	TGCGGCAATTCCTCGACA	+	2764; hypothetical protein
2981974	4.93	GGTTACGGCCCCATCACA	−	2790; CarD family transcriptional regulator

3009135	4.19	TTTGCGGTCCGTTCGACA	−	*ibpA*; small heat shock protein IbpA
3040855	4.32	CGTCCAGATCGTGGCACC	+	*dorS*; DMSO/TMAO-sensor hybrid histidine kinase (EC:2.7.13.3)
3090723	4.55	TGCCCGCAGAATGCGACA	−	*cspA3*; cold shock protein CspA
3129189	2.69		−	2938; hypothetical protein
3161881	2.23	TGCCTCCGCACGCGGACA	+	*atpH*; ATP synthase F1 subunit delta (EC:3.6.3.14)
3266484	5.87	TGCCGCAGATGCGCGACA	−	3072; hypothetical protein
3271018	3.97		+	3078; polysaccharide biosynthesis/export family protein
3308012	5.02	TGCCGGACATGCCTTTCA	+	*gcvT2*; glycine cleavage T protein (EC:2.1.2.10)
3310289	7.08	TGCTCTCTGGCCGACACA	+	3113; hypothetical protein
3321695	3.26		+	3125; heavy metal transport/detoxification protein family
3323324	5	TGTCGGGCAACAACATCA	−	*rpmB*; 50S ribosomal protein L28
3338749	3.6		+	*cspD*; cold shock-like protein CspD
3360664	4.16	TGGTGAACACCTCGGCCA	−	3162; mandelate racemase/muconate lactonizing enzyme family protein
3379752	5.18	TGTTTCTTGCGAAGTTCA	+	3181; hypothetical protein
3386212	5.09	TGTGTTGGTACAGTTATA	−	3187; AlgR/AgrA/LytR family transcriptional regulator
3392775	2.65	TGCCGAGCAGGTTGAACA	−	*ftsH*; cell division protease FtsH (EC:3.4.24.-)
3415390	2.73		+	3214; hypothetical protein
3499285	4.71	TGTTGCCCCCGGGTCACC	−	*dapE*; succinyl-diaminopimelate desuccinylase (EC:3.5.1.18)
3512465	3.09	GGTCGATCTCGCCCGACA	+	*spoT*; bifunctional alarmone synthase/hydrolase (EC:2.7.6.5)
3515627	3.32	CGTCGGTGTTGGAAAACA	+	3326; lipoprotein
3548710	4.38		+	*metH1*; methionine synthase subunit A (EC:2.1.1.13)
3573414	3.02	TGTAATCTACTGCCTGCA	−	3377; hypothetical protein
3605037	4.88		−	3403; hypothetical protein
3631736	9.45	TGTAAACTAAAATGGACA	+	*livM3*; branched-chain amino acid ABC transporter permease LivM
3644584	5.03	AGTTGGATTTTCCGGACA	−	3441; hypothetical protein
3714536	6.65		−	*cbiX*; cobalamin biosynthesis protein CbiX
3718186	4.28	TGCGGCAAAATGAAGACA	−	3510; hypothetical protein
