# Supplementary material for: The Vitamin B12-Dependent Photoreceptor AerR Relieves Photosystem Gene Repression by Extending the Interaction of CrtJ with Photosystem Promoters
Source: mBio. 2017 Mar 21;8(2):e00261-17. doi: 10.1128/mBio.00261-17 (PMC5362033; doi:10.1128/mBio.00261-17)
Supplement: TABLE S5 [file mbo002173237st5.pdf]

Table S5. Unique and common CrtJ binding sites under two different growth conditions.

	Binding sites
**Shared by both conditions**	*ldc*; lysine/ornithine decarboxylase (EC:4.1.1.17 4.1.1.18)
	dacC1; D-alanyl-D-alanine carboxypeptidase (EC:3.4.16.4)
	1272; hypothetical protein
	rpmB; 50S ribosomal protein L28
	nuoA; NADH-quinone oxidoreductase subunit A (EC:1.6.99.5)
	mraZ; protein MraZ
	atpB; ATP synthase F0 subunit A (EC:3.6.3.14)
	ctrA; cell cycle transcriptional regulator CtrA
	bchC; 2-desacetyl-2-hydroxyethyl bacteriochlorophyllide A dehydrogenase
	871; hypothetical protein
	dnaA; chromosomal replication initiator protein DnaA
	1311; hypothetical protein
	1823; LuxR family autoinducer-binding transcriptional regulator
	clpX; ATP-dependent Clp protease ATP-binding subunit ClpX (EC:3.4.21.92)
	rplU; 50S ribosomal protein L21
	259; porin family protein
	nrdJ1; ribonucleoside-diphosphate reductase NrdJ (EC:1.17.4.1)
	118; sigma 54 modulation protein/ribosomal protein S30EA
	thiN; thiamine pyrophosphokinase (EC:2.7.6.2)
	livM3; branched-chain amino acid ABC transporter permease LivM
	rpsO; 30S ribosomal protein S15
	1277; RNA-directed DNA polymerase (EC:2.7.7.49)
	rpmF; 50S ribosomal protein L32
	cspA1; cold shock protein CspA
	dapE; succinyl-diaminopimelate desuccinylase (EC:3.5.1.18)
	2938; hypothetical protein

2566; reverse transcriptase catalytic domain-containing protein (EC:2.7.7.49)
ihfA; integration host factor subunit alpha
metG; methionyl-tRNA synthetase (EC:6.1.1.10)
cspA3; cold shock protein CspA
1900; hemolysin-type calcium-binding repeat family protein (EC:4.6.1.1)
3162; mandelate racemase/muconate lactonizing enzyme family protein
3187; AlgR/AgrA/LytR family transcriptional regulator
3125; heavy metal transport/detoxification protein family
aglE; alpha-glucoside ABC transporter substrate-binding protein
crtD; methoxyneurosporene dehydrogenase (EC:1.14.99.-)
3072; hypothetical protein
ddl; D-alanine--D-alanine ligase (EC:6.3.2.4)
mcpA1; methyl-accepting chemotaxis protein McpA
spoT; GTP diphosphokinase/guanosine-3',5'-bis(diphosphate) 3'-pyrophosphohydrolase (EC:2.7.6.5)
bchF; 2-vinyl bacteriochlorophyllide hydratase (EC:4.2.1.-)
2634; response regulator receiver modulated diguanylate cyclase/phosphodiesterase
gidA; tRNA uridine 5-carboxymethylaminomethyl modification enzyme GidA
metH1; methionine synthase subunit A (EC:2.1.1.13)
uvrC; UvrABC system protein C
212; hypothetical protein
rpmG; 50S ribosomal protein L33
1452; acriflavin resistance protein family
2006; hypothetical protein
ccoN; cbb3-type cytochrome c oxidase subunit I (EC:1.9.3.1)
1682; hypothetical protein
hemE; uroporphyrinogen decarboxylase (EC:4.1.1.37)
1874; hypothetical protein
rne; ribonuclease E (EC:3.1.4.-)
hemN2; oxygen-independent coproporphyrinogen-III oxidase (EC:1.3.99.22)
185; hypothetical protein

	pucB; light-harvesting protein B-800/850 subunit beta
	1209; cell wall hydrolase, SleB
	pccB; propionyl-CoA carboxylase subunit beta (EC:6.4.1.3)
	1081; group 1 glycosyl transferase (EC:2.4.1.-)
	gppA; guanosine-5'-triphosphate,3'-diphosphate pyrophosphatase (EC:3.6.1.40)
	crtA; spheroidene monooxygenase
	3113; hypothetical protein
	1448; hypothetical protein
	bchE; magnesium-protoporphyrin IX monomethyl ester anaerobic oxidative cyclase (EC:1.14.13.81)
	gcvT2; glycine cleavage T protein (EC:2.1.2.10)
	1268; hypothetical protein
	gshB; glutathione synthase (EC:6.3.2.3)
	2005; hypothetical protein
	kup; potassium transporter
	ftsZ; cell division protein FtsZ
	2790; CarD family transcriptional regulator
	potA1; polyamine ABC transporter ATP binding protein PotA (EC:3.6.3.31)
	1919; membrane protein involved in aromatic hydrocarbon degradation
	2119; type 12 family methyltransferase (EC:2.1.1.-)
	1845; hypothetical protein
	acpP1; acyl carrier protein
	3441; hypothetical protein
	atpH; ATP synthase F1 subunit delta (EC:3.6.3.14)
	2037; CbiM family cobalamin biosynthesis protein
	nusG; transcription antitermination protein NusG
**Photosynthetic specific**	*rpmH*; 50S ribosomal protein L34
	2154; TM2 domain-containing protein
	548; BolA family protein

1276; Cas1 family CRISPR-associated protein
pufQ; cytochrome, subunit PufQ
purL; phosphoribosylformylglycinamidine synthase II (EC:6.3.5.3)
434; hypothetical protein
hupD; hydrogenase maturation protease HupD (EC:3.4.23.-)
prfC; peptide chain release factor 3
hupA; hydrogenase small subunit (EC:1.12.99.6)
cbiX; cobalamin biosynthesis protein CbiX
542; hypothetical protein
79; iojap-related protein
980; phage virion morphogenesis protein
phbC; poly(3-hydroxyalkanoate) polymerase (EC:2.3.1.-)
1579; hypothetical protein
cspD; cold shock-like protein CspD
3326; lipoprotein
3078; polysaccharide biosynthesis/export family protein
gcvA; glycine cleavage system transcriptional activator
hvrB; AHCY transcriptional activator HvrB
424; hypothetical protein
dksA2; DnaK suppressor protein
3403; hypothetical protein
rpsL; 30S ribosomal protein S12
fusA2; translation elongation factor G (EC:3.6.5.3)
1746; hypothetical protein
2161; hypothetical protein
3181; hypothetical protein
416; LysR family transcriptional regulator
xylF; xylose ABC transporter xylose-binding protein XylF
2326; phage integrase
910; hypothetical protein

**Aerobic specific**	2415; hypothetical protein
	mdoH; glucans biosynthesis glucosyltransferase H (EC:2.4.1.-)
	rhlE; ATP-dependent RNA helicase RhlE (EC:3.6.1.-)
	cspA2; cold shock protein CspA
	2075; PAS/PAC sensor domain-containing protein
	glnB1; nitrogen regulatory protein P-II
	puhA; photosynthetic reaction center subunit H
	ftsH; cell division protease FtsH (EC:3.4.24.-)
	dorS; DMSO/TMAO-sensor hybrid histidine kinase (EC:2.7.13.3)
	dnaK; chaperone DnaK
	3214; hypothetical protein
	901; hypothetical protein
	lpxC; UDP-3-O-[3-hydroxymyristoyl] N-acetylglucosamine deacetylase (EC:3.5.1.-)
	711; universal stress family protein
	3377; hypothetical protein
	ibpA; small heat shock protein IbpA
	2764; hypothetical protein
	3510; hypothetical protein
	1330; hypothetical protein
	642; peptidoglycan binding domain-containing protein
	842; hypothetical protein
	pyrG; CTP synthase (EC:6.3.4.2)
	corC; magnesium and cobalt efflux protein CorC
	2670; Fur family transcriptional regulator
	bdhA; 3-hydroxybutyrate dehydrogenase (EC:1.1.1.30)
	873; XRE family transcriptional regulator
	flgB; flagellar basal-body rod protein; FlgB
	479; COQ9 family ubiquinone biosynthesis protein
	rpsF; 30S ribosomal protein S6
	rplL; 50S ribosomal protein L7/L12

1010; DNA binding protein
adhC; bifunctional alcohol dehydrogenase/S-(hydroxymethyl)glutathione dehydrogenase (EC:1.1.1.1)
1463; hypothetical protein
2495; GTPase, EngC family (EC:3.6.1.-)
hsdR1; type I restriction-modification system RcaSBIP subunit R (EC:3.1.21.3)
hvrA; trans-acting regulatory protein HvrA
gst; glutathione S-transferase (EC:2.5.1.18)
rplO; 50S ribosomal protein L15
flaA; flagellin protein
ccoG; cbb3-type cytochrome c oxidase accessory protein CcoG
gcvT1; glycine cleavage T protein (EC:2.1.2.10)
930; hypothetical protein
sufB; FeS assembly protein SufB
rpsM; 30S ribosomal protein S13
1234; hypothetical protein
