# Supplementary material for: The Vitamin B12-Dependent Photoreceptor AerR Relieves Photosystem Gene Repression by Extending the Interaction of CrtJ with Photosystem Promoters
Source: mBio. 2017 Mar 21;8(2):e00261-17. doi: 10.1128/mBio.00261-17 (PMC5362033; doi:10.1128/mBio.00261-17)
Supplement: FIG S1 [file mbo002173237sf1.pdf]

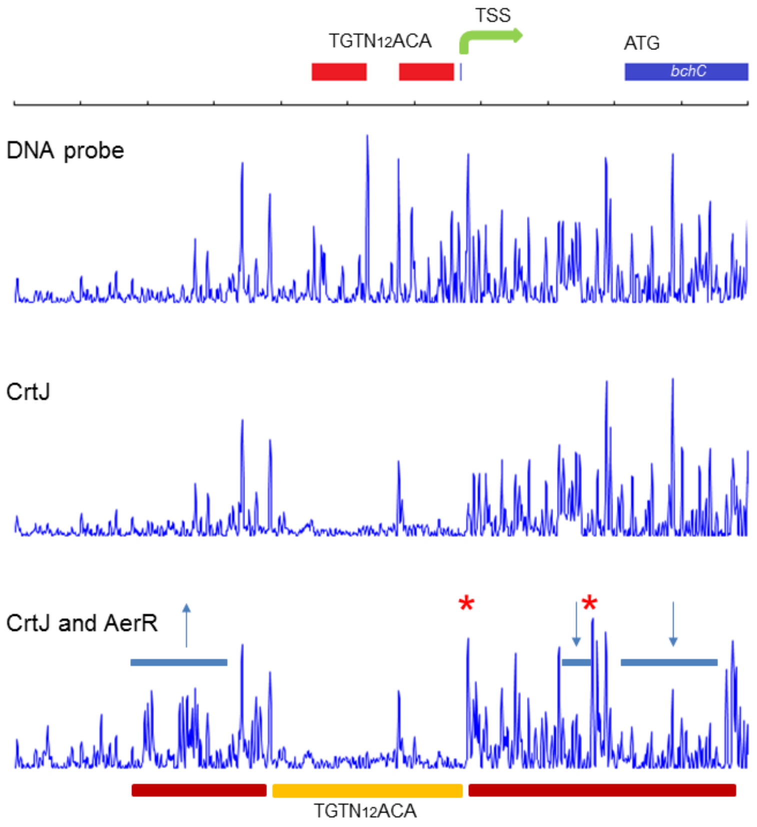

Figure S1. DNase I footprint analysis of $CrtJ_2$ and AerR-$CrtJ_2$- binding to the *bchC* promoter. Top panel; limited DNase I digestion of the *bchC* promoter DNA probe. Middle panel; limited DNase I digestion of the DNA probe that was preincubated with $CrtJ_2$ with the protected region highlighted with a yellow bar. Bottom panel; limited DNase I digestion of the DNA probe that was preincubated with AerR-$CrtJ_2$ complex. The extended protection region is highlighted with red bars. Areas of DNase I suppression in the extended area are highlighted with blue down arrows above blue bars while areas of individual base hypersensitivity are highlighted with a red asterisk and areas of extended hypersensitivity have a bar with an upward blue arrow. The positions of the two $TGTN_{12}ACA$ palindromes, transcription start site (TSS) and the start codon of *bchC* gene are labeled accordingly.
