# Supplementary material for: The Vitamin B12-Dependent Photoreceptor AerR Relieves Photosystem Gene Repression by Extending the Interaction of CrtJ with Photosystem Promoters
Source: mBio. 2017 Mar 21;8(2):e00261-17. doi: 10.1128/mBio.00261-17 (PMC5362033; doi:10.1128/mBio.00261-17)
Supplement: FIG S2 [file mbo002173237sf2.pdf]

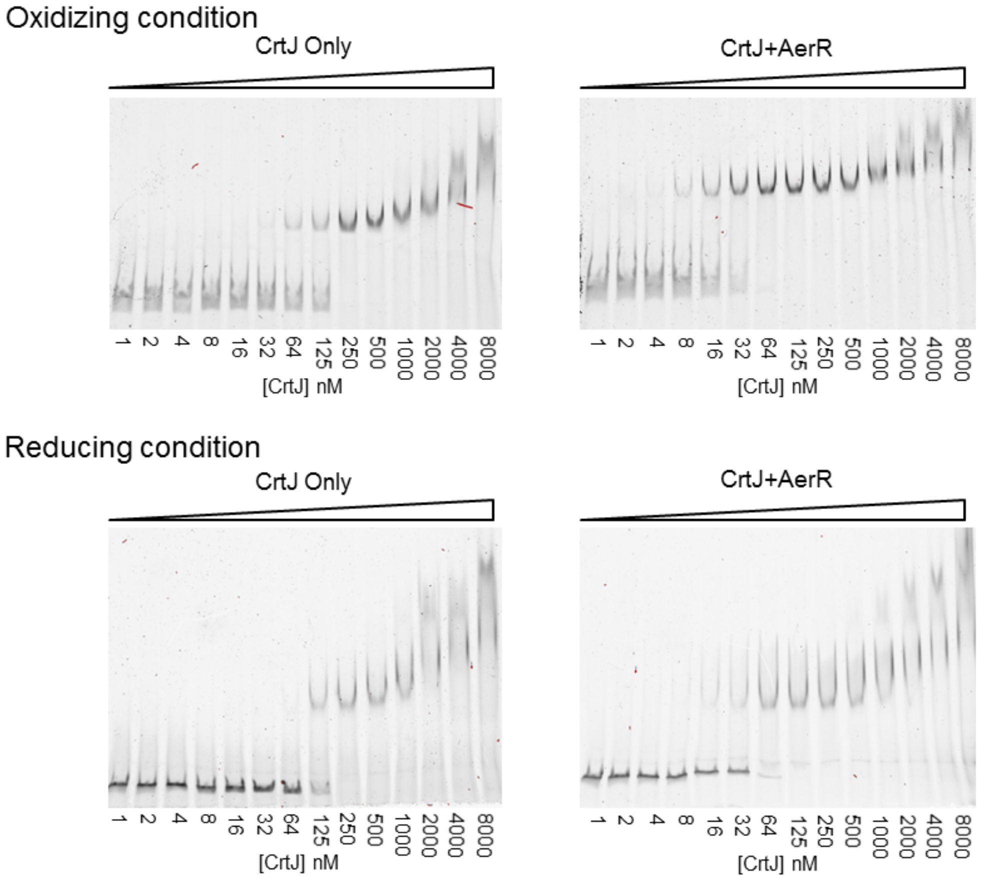

Figure S2. Gel mobility shift assay with CrtJ and AerR under both oxidizing and reducing conditions. 10 nM *bchC* promoter DNA fragment was incubated with various concentrations of purified CrtJ. 10 μM AerR and 10 mM DTT was added to the reactions accordingly. With AerR, the DNA probe can be shifted at lower CrtJ concentration under both oxidizing and reducing conditions.
