# Supplementary material for: The Vitamin B12-Dependent Photoreceptor AerR Relieves Photosystem Gene Repression by Extending the Interaction of CrtJ with Photosystem Promoters
Source: mBio. 2017 Mar 21;8(2):e00261-17. doi: 10.1128/mBio.00261-17 (PMC5362033; doi:10.1128/mBio.00261-17)
Supplement: TEXT S1 [file mbo002173237s1.pdf]

Supplemental Materials and Methods

Strains and growth conditions

Rhodobacter capsulatus SB1003 was used as the wild-type parental strain with the *ΔaerR, ΔcrtJ* and a *ΔaerR ΔcrtJ* double deletion strains derived from SB1003 as described previously (1). *Escherichia coli* BL21 (DE3) (2) was used to overexpress protein with *E. coli* S17-1 λpir (1) used to introduce different plasmids to *R. capsulatus* via conjugation. *R. capsulatus* strains were routinely grown in PY salts liquid medium at 34 °C (2). Under aerobic conditions, cells were grown as 8 ml cultures in 50 ml Erlenmeyer flasks shaken at 400 rpm in dark to a scattering optical density at 660 nm of 0.3. Under photosynthetic conditions, cells were grown in 8 ml screw capped tubes filled with PY medium that were illuminated with a 60W tungsten light source. *E. coli* strains were routinely grown in LB medium at 37 °C. When used for plasmid selection, antibiotics were used at 1 μg/ml gentamicin in PY medium while in LB medium gentamicin was used at 10 μg/ml and kanamycin at 50 μg/ml.

Plasmid and strain construction

Suicide plasmid pZJD29a was used to generate a non-polar in-frame deletion and chromosomal tagged strain in *Rhodobacter capsulatus* SB1003 as previously reported (3). pSRKGm (4) was used to express 3xFLAG-tagged CrtJ in *R. capsulatus* and pSUMO was used for overexpression of proteins in *E. coli* (2). For construction of 3xFLAG-CrtJ, oligonucleotide containing a 3xFLAG sequence was constructed that fused to first 21 nucleotide of *crtJ* gene as a forward primer. The amplified 3xFLAG-tagged *crtJ* was cloned and inserted between the NdeI and SacI sites on pSRKGm with the resulting

plasmid construct conjugated to Δ*crtJ* strain and a Δ*aerR* Δ*crtJ* strain for ChIP experiments. In frame clean deletion of CrtJ from the chromosome was made by constructing a suicide plasmid that contained 500 bp upstream of CrtJ through the 3rd codon of CrtJ and three codons upstream of the CrtJ stop codon to 500 bp downstream of CrtJ connected by crossover PCR. A double recombination event with this suicide involving initial GmR followed by selective growth on sucrose containing plates as has been described previously (1). For construction of a chromosomally encoded 3xV5 CrtJ, a DNA fragment contained 500 bp upstream of CrtJ and 500 bp into CrtJ open reading frame was constructed that also contained a 3xV5 tag inserted upstream of the first codon of CrtJ. This plasmid was constructed by first PCR amplifying and cloning the upstream 500 bp fragment using a reverse primer that contained 3xV5 sequence. A 500 bp region of the CrtJ coding sequence was also amplified with the same 3xV5 sequence in the forward primer. These two fragments were subsequently connected using crossover PCR and inserted into the pSRKGm suicide plasmid. Wild type strain was used as parental strain with recombination of this construct into the chromosome involving initial selection for GmR followed by growth on sucrose selective medium. The successful deletion of *crtJ* was confirmed by sequencing, and the successful insertion of the V5-tag was confirmed by sequencing and Western blot analysis.

Construction of an Δ*aerR* Δ*crtJ* double deletion strain was constructed as a double recombination event as described above for the *crtJ* deletion strain with the exception that the first 3 codons of *aerR* were fused to the last three codons of *crtJ*. A 3xFLAG-AerR construct was made that placed the FLAG epitope at the C-terminus of AerR. This 3xFLAG epitope was fused on a reverse primer and the AerR-3xFLAG was cloned into

the NdeI and SacI sites on pSRKGm as described for CrtJ. The establishment of functional activity of the CrtJ and AerR FLAG constructs is provided in a Supplemental Complementation Data File.

ChIP-seq

Two epitope-tagged CrtJ constructs (N-terminal 3×FLAG and N-terminal 3×V5) were used in the experiments. In one set of experiments FLAG-tagged CrtJ was expressed from pSRKGm plasmid in three independent biological replicates by the addition of 100 μM IPTG added 30 min before formaldehyde cross-linking. In a second set of experiments, V5-tagged CrtJ was recombined into the chromosome at its native location from which it was expressed form its native promoter. Two biological replicates of the V5-tagged CrtJ were subsequently formaldehyde cross linked and immunoprecipitated. ChIP-seq analysis of AerR involved the use of a C-terminal 3×FLAG-tagged AerR construct expressed from pSRKGm with no IPTG induction.

ChIP was performed by initiating crosslinking by the addition of 1% formaldehyde to a 50 ml culture (OD_{660} 0.3-0.4) for 20 min at room temperature. Cross-linking was then stopped by the addition of glycine to a final concentration of 0.125 M for 5 min. Cells were centrifuged and washed twice with cold TBS (50 mM Tris-HCl pH 8.0, 150 mM NaCl) before being resuspened in 4 ml cold FA-M2 buffer (50 mM Tris-HCl pH 8.0, 150 mM NaCl, 1 mM EDTA, 1% Triton X-100) for FLAG-tagged cells or in RIPA buffer as described by Davies et al. (5), for V5-tagged cells. The cells were lysed by passage twice through a French Press at 18,000 psi followed by sonication 5 times for 30 s with needle sonication (Misonix). The cell lysate was clarified by centrifugation and the supernatant

was collected for immunoprecipitation with the exclusion of 50 μl of lysate that was saved from which DNA was purified as input DNA. 100 μl ANTI-FLAG M2 or ANTI-V5 affinity agarose gel (Sigma) was added to the lysate and incubated overnight with rotation at 4 °C. The beads were gently centrifuged and supernatant was removed carefully followed by washing 5× with 1 ml FA-M2 (RIPA buffer for V5-tagged sample) and 3× with TBS for 15 min. The tagged protein was eluted from the beads by adding 200 μl elution buffer (50 mM Tris-HCl pH 8.0, 10 mM EDTA, 1% SDS) and incubating at 65°C for 30 min. Supernatant was collected and incubated overnight with 5 μl of 20 mg/ml Proteinase K at 65°C. Same volume of phenol/chloroform/isopropanol (25:24:1) was added followed by centrifugation. The upper phase was collected to which the same volume of chloroform was added followed by centrifugation. The ~ 200 μl upper phase was collected and 1/10 volume of 3 M sodium acetate (pH 5.2), 2.5 volume of pure ethanol and 2 μl GlycoBlue (ThermoFisher Scientific) were added. The mixture was precipitated at -20 °C overnight and then centrifuged at 4 °C for 15 min. The pellet was washed with cold 75% ethanol and dried at room temperature for 10 min. 20 μl nuclease free water was used to resuspend the DNA pellet. The concentration and quality of the DNA was checked in 2200 TapeStation using High Sensitivity D1000 ScreenTape (Agilent Technologies).

Library construction and sequencing were performed by University of Wisconsin Madison Biotechnology Center DNA Sequencing Facility. Both immunoprecipitated and input DNA were processed using TruSeq ChIP Sample Preparation Kit (Illumina) with minor modifications. Libraries were size selected for an average size of 350 bp using SPRI beads based selection. Single end sequencing reactions (100×) were performed on

Illumina HiSeq2000 sequencer with raw sequence read files deposited in Sequence Read Archive (SRA) with the accession number SRP082587. The raw reads were trimmed with Trimmomatic (6) and aligned to genome with Bowtie2 (7). SAM files were converted to BAM files and subjected to MACS (Model-based Analysis of ChIP-Seq) program (8). Wiggle files were visualized in Integrative Genomics Viewer (IGV) (9). CrtJ binding peaks called as significant binding sites if they were identified as such by MACS in both the CrtJ-FLAG and CrtJ-V5 constructs and were present in 3 of 5 biologically independent replicates.

ChIP-exo

Cells used for ChIP-exo were grown, collected, fixed and lysed as described in ChIP-seq experiments. ChIP-exo procedures were performed as previously reported (10). Oligonucleotides for P7 exo-adapter and P5 exo-adaptor were synthesized by IDT Corp. Adaptors were obtained by mixing 40 μM of complement oligonucleotides in Tris-NaCl buffer (10 mM Tris-HCl pH 7.5, 10 mM NaCl) and annealed by heating in a thermocycler to 97 °C followed by a stepdown at the rate of -1 °C for 72 cycles. The adaptors were further diluted to 15 μM in same Tris-NaCl buffer to form a working stock. For each ChIP-exo replicate, 4 ml of cell lysate was divided into 2 aliquots for further immunoprecipitation and reactions with higher efficiency. In each tube, 50 μl ANTI-FLAG M2 affinity agarose gel was added. The tubes were incubated on an end-to-end rotator overnight at 4 °C. After incubation, the beads were gently centrifuged with the supernatant removed followed by washing the beads 5× with 1 ml FA-M2 and 3× with 1 ml TBS. Next, 5 different enzymatic reactions mixture was added to the beads followed

with 5× FA-M2 and 3× TBS washes between each reaction. These reactions were: *End repair;* comprised of 1 µl ATP (100 mM), 1 µl dNTP (10 mM each), 5 µl T4 DNA polymerase (3 U/µl), 1 µl DNA polymerase I Klenow (5 U/µl), 10 µl T4 polynucleotide kinase (10 U/µl), 10 µl NEBuffer 2(10×) in total 100 µl at 30 °C for 1 h. *Ligation of the P7 exo-adaptor*; 1 µl ATP (100 mM), 10 µl P7 exo-adaptor (15 µM), 1 µl T4 DNA ligase (2000U/µl), 10 µl NEBuffer 2 (10×) in total 100 µl at 16 °C for 1 h. *Nick repair*; 1.5 µl dNTP (10 mM each), 1.5 µl phi29 DNA polymerase (10 U/µl) in 100 µl 1× phi29 reaction buffer (50 mM Tris-HCl pH, 10 mM $MgCl_2$, 10 mM $(NH_4)_2SO_4$, 1 mM DTT). *Lambda exonuclease digestion;* 2 µl Lambda exonuclease (5 U/µl), 10 µl Lambda exonuclease buffer (10×) in total 100 µl at 37 °C for 1 h. *$RecJ_f$ exonuclease digestion*; 1 µl $RecJ_f$ exonuclease (30 U/µl), 10 µl NEBuffer 2 (10×) in total 100 µl at 37 °C for 1 h. All the enzymes and buffers were obtained from NEB except phi29 buffer. After exonuclease digestion the protein-DNA complex was eluted from the beads by adding 100 µl elution buffer and incubating at 65 °C for 30 min. Another 100 µl elution buffer was added and kept at 65 °C for 30 min for better elution. 200 µl elution buffer containing protein-DNA complex was reverse cross-linked and precipitated as described in ChIP-seq experiments. The DNA pellet was resuspended in 20 µl nuclease-free water, and heated to 95 °C for 5 min to denature the DNA. 1 µl P7 primer (5 µM), 5 µl phi29 buffer (NEB, 10×) and 22 µl nuclease-free water were immediately added to make the final volume 48 µl. The mixture was further incubated at 65 °C for 5 min to anneal P7 primer to the DNA. Then the mixture was cooled down to 30 °C and 1 µl phi29 polymerase and 1 µl dNTP (10 mM each) were added to the reaction and then incubated for another 30 min. The primer extension reaction was stopped by heating at 65 °C for 10 min. 90 µl AMPure XP beads

were added to the reaction and the beads were washed twice with cold 80% ethanol before adding 20 µl elution buffer (10 mM Tris-Acetate, pH 8.0). P5 exo-adapter was added to the DNA by adding 1 µl P5 exo-adaptor (15 µM), 1 µl T4 DNA ligase (2000U/µl), 5 µl T4 DNA ligase buffer (10×) and 23 µl nuclease-free water to the 20 µl eluent and incubating for 1 h at 16 °C. The DNA was cleaned up with AMPure beads and eluted in 20 µl again.

For library construction, primers with indexes were used to amplify the DNA. 2.5 µl of each primer (10 µM) and 25 µl NEBNext High-Fidelity 2× PCR Master Mix were added to DNA sample. 15 PCR cycles (10 s at 98 °C, 30 s at 65 °C, 30 s at 72 °C) were used to amplify the library followed by AMPure beads cleanup. 20 µl elution buffer was used to elute DNA from two tubes of the same replicate to recombine the library. The quality of the library was analyzed in 2200 TapeStation using D1000 ScreenTape (Agilent Technologies). Sequencing of the DNA libraries was performed by the Center for Genomics and Bioinformatics at Indiana University Bloomington using MiSeq system (Illumina) for 50 cycles of single end sequencing.

For data analysis, the raw reads were trimmed and aligned to genome as described above. SAM files were converted to BAM files and subjected to MACE (Model-based Analysis of ChIP-Exo) program (11). Forward and reverse border containing files were visualized in IGV.

RNA isolation and RNA-seq

Rhodobacter capsulatus strains were grown to early exponential phase (OD_{660} 0.3-0.35) before collection. 1.5 ml of cell cultures were harvested and stored at -80 °C until needed.

For each strain under each condition, triplicates were used. Total RNA was extracted using ISOLATE II RNA Mini Kit (Bioline) followed by TURBO DNase (Ambion) treatment. The reaction mixture was cleaned up and concentrated by RNeasy MinElute Cleanup Kit (QIAGEN) and assayed for DNA contamination by performing PCR amplification on samples with verses without reverse transcriptase treatment. Final RNA concentrations were measure by NanoDrop (Thermo Scientific). Further quality control was performed in 2200 TapeStation using RNA ScreenTape (Agilent Technologies). Library construction and RNA-sequencing were performed by University of Wisconsin Madison Biotechnology Center DNA Sequencing Facility. Generally, ribosomal RNA was depleted from 2 μg total RNA using an EpiCentre Ribo-Zero Magnetic (Bacteria) kit. Illumina mRNA-Seq libraries were constructed using TruSeq RNA Sample Prep kit (Illumina) according to manufacturer's protocol. Single end sequencing reactions (100×) were performed on Illumina HiSeq2000 sequencer with raw sequence read files deposited in Sequence Read Archive (SRA) with the accession number SRP082587.

The raw reads were trimmed and aligned to the genome as described above. HTSeq-count (12) was used to count read numbers in each gene followed by differential expression analysis using DESeq2 package in R (13).

Protein purification

pSUMO-AerR or pSUMO-CrtJ bearing *E. coli* BL21 (DE3) strains were used to overexpress SUMO-tagged AerR or CrtJ according to previous studies (1, 2). In brief, after overnight induction at 16 °C, cells were collected and lysed by French Press. The lysate was clarified and loaded onto HisTrap column (GE) followed by elution of

imidazole gradient. SUMO-AerR and SUMO-CrtJ fractions were collected and digested by SUMO protease. Tag-less AerR and CrtJ were further purified by size exclusion chromatography. The fractions containing desired protein were collected and concentrated. The final protein concentrations were measured by Bradford assay (Advanced Protein Assay Reagent, Cytoskeleton).

Gel Mobility Shift Assay

A 325 bp segment of the *bchC* promoter region was amplified and purified as reported (1). The DNA concentration was measured by NanoDrop (Thermo Scientific). 10 nM *bchC* promoter DNA fragment was incubated with various concentrations of purified CrtJ in binding buffer containing 25 mM Tris-HCl pH 8.0, 150 mM NaCl, 2 mM $MgCl_2$, 10% glycerol and 0.6 mg/ml heparin. 10 µM AerR and 10 mM DTT was added to the reactions when needed. The 10 µl reaction mixtures were incubated at room temperature (23 °C) for 10 min before loaded to a 4% native gel and electrophoresed at 4 °C in 50 mM Tris-HCl pH 8.0, 380 mM glycine and 2 mM EDTA. The gel was then stained with SYBR Green dye (Invitrogen) and visualized in Gel Doc XR+ system (BioRad). The gel band intensities were quantified with Fiji, and the binding percentage data were fitted using Hill equation in Origin 9.0.

DNase I Footprint Assay

The *bchC* promoter region was prepared as described above with the exception that the DNA segment was amplified using a 5-HEX labeled forward primer and a 6-FAM labeled reverse primer. 20 nM *bchC* promoter DNA fragment was incubated with various CrtJ

concentrations in 20 µl binding buffer for 10 min followed by a 15 min DNase I digestion that was started by addition of 5 µl 1:128 diluted DNase I (NEB). The reaction was stopped by adding of same volume of 0.5 M EDTA (pH 8.0). DNA fragments were purified by MinElute PCR purification kit (QIAGEN) in a final volume of 15 µl. 0.2 µl 500 LIZ Size Standard (Applied Biosystem) was added before transferring the samples to 96-well plate. The plate was heated to 95°C for 5 min and immediately cooled on ice to generate single strand DNA. The fluorescent single strand DNA fragments were separated and detected with a 3730 DNA analyzer (Applied Biosystem). The results were further analyzed using Peak Scanner Software v1.0 (Applied Biosystem).

Multi-Angle Light Scattering

The size of AerR-CrtJ complex was determined by size exclusion chromatography coupled with multi-angle light scattering (SEC-MALS, WYATT Technology Corp). Various concentrations of AerR and CrtJ in 100 µl binding buffer were loaded to a prepacked Superdex 200 column (10×300 mm, GE). The protein was separated with 25 mM Tris-HCl pH 8.0, 150 mM NaCl and 5% glycerol at 0.3 ml/min. The elution flow was coupled to multi-angle static light scattering analysis and refractive index measurement. The light scattering results were analyzed using ASTRA 6 (WYATT technology) with BSA for calibration.

References

1. Cheng Z, Li K, Hammad LA, Karty JA, Bauer CE (2014) Vitamin B_{12} regulates photosystem gene expression via the CrtJ antirepressor AerR in Rhodobacter capsulatus. Mol Microbiol 91: 649-664.
2. Cheng Z, Wu J, Setterdahl A, Reddie K, Carroll K, et al. (2012) Activity of the

tetrapyrrole regulator CrtJ is controlled by oxidation of a redox active cysteine located in the DNA binding domain. Mol Microbiol 85: 734-746.
3. Masuda S, Bauer CE (2004) Null mutation of HvrA compensates for loss of an essential relA/spoT-like gene in *Rhodobacter capsulatus.* J Bacteriol 186: 235-239.
4. Khan SR, Gaines J, Roop RM, 2nd, Farrand SK (2008) Broad-host-range expression vectors with tightly regulated promoters and their use to examine the influence of TraR and TraM expression on Ti plasmid quorum sensing. Appl Environ Microbiol 74: 5053-5062.
5. Davies BW, Bogard RW, Mekalanos JJ (2011) Mapping the regulon of *Vibrio cholerae* ferric uptake regulator expands its known network of gene regulation. Proc Natl Acad Sci U S A 108: 12467-12472.
6. Bolger AM, Lohse M, Usadel B (2014) Trimmomatic: a flexible trimmer for Illumina sequence data. Bioinformatics 30: 2114-2120.
7. Langmead B, Salzberg SL (2012) Fast gapped-read alignment with Bowtie 2. Nat Methods 9: 357-359.
8. Zhang Y, Liu T, Meyer CA, Eeckhoute J, Johnson DS, et al. (2008) Model-based analysis of ChIP-Seq (MACS). Genome Biol 9: R137.
9. Robinson JT, Thorvaldsdottir H, Winckler W, Guttman M, Lander ES, et al. (2011) Integrative genomics viewer. Nat Biotechnol 29: 24-26.
10. Serandour AA, Brown GD, Cohen JD, Carroll JS (2013) Development of an Illumina-based ChIP-exonuclease method provides insight into FoxA1-DNA binding properties. Genome Biol 14: R147.
11. Wang L, Chen J, Wang C, Uuskula-Reimand L, Chen K, et al. (2014) MACE: model based analysis of ChIP-exo. Nucleic Acids Res 42: e156.
12. Anders S, Pyl PT, Huber W (2015) HTSeq--a Python framework to work with high-throughput sequencing data. Bioinformatics 31: 166-169.
13. Love MI, Huber W, Anders S (2014) Moderated estimation of fold change and dispersion for RNA-seq data with DESeq2. Genome Biol 15: 550.
