# Supplementary material for: The Vitamin B12-Dependent Photoreceptor AerR Relieves Photosystem Gene Repression by Extending the Interaction of CrtJ with Photosystem Promoters
Source: mBio. 2017 Mar 21;8(2):e00261-17. doi: 10.1128/mBio.00261-17 (PMC5362033; doi:10.1128/mBio.00261-17)
Supplement: TEXT S2 [file mbo002173237s2.pdf]

Supplemental Functional Complementation Data

To express CrtJ under physiological concentration, different concentrations of IPTG were added to Δ*crtJ* pSRKGm::3xFLAG-CrtJ strain and the protein levels were compared with chromosomal 3xFLAG-CrtJ by Western Blot. 100 μM IPTG was chosen because it gave comparable protein expression level for CrtJ (FCD Fig 1A). Further more, the transcript level of plasmid-borne CrtJ with 100 μM IPTG were compared with that in wild type by RNA-seq. The result showed that under this condition, CrtJ transcript levels were also close to wild type (FCD Fig 1B).

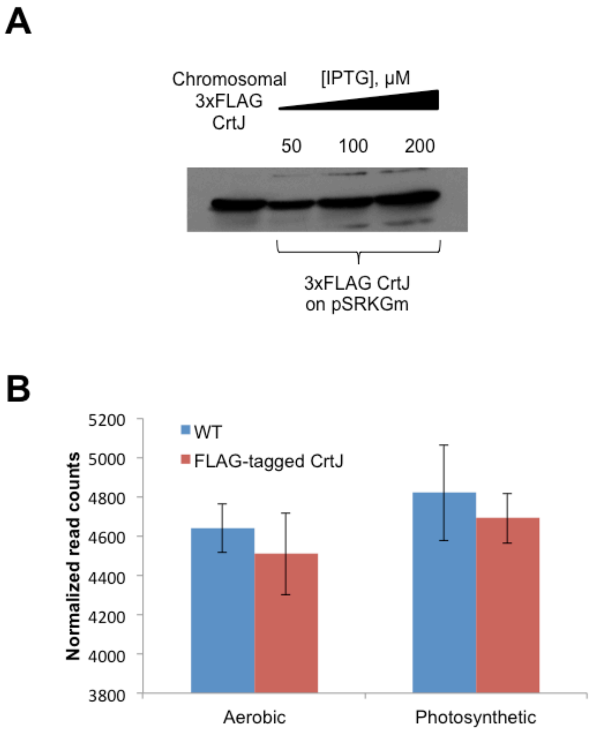

FCD Fig 1. A) Western blot result showing different expression levels of 3xFLAG CrtJ upon addition of IPTG at different concentrations. B) Normalized read counts on *crtJ* gene from RNA-seq results under 100 μM IPTG.

To check if 3xFLAG-tagged CrtJ still retains its biological function, spectral analyses on crude cell extract were performed with normalization as described before [1]. CrtJ functions as an aerobic repressor of photosystem. When wild type cells were grown aerobically, very little amount of photosystem would be produced; however in Δ*crtJ* strain, a notable absorption peak in the infrared region could be seen that showed the expression of photosystem, indicating the loss of CrtJ function (FCD Fig 2A). In the Δ*crtJ* pSRKGm::3xFLAG-CrtJ strain (grown with 100 μM IPTG), the spectrum was almost the same as wild type spectrum, suggesting the 3xFLAG-CrtJ still retains its function. RNA-seq analysis on these strains revealed that 3xFLAG-CrtJ retained more than 80% of the activity based on its repression on photosynthesis gene (*bchC*) expression (FCD Fig 2B)

A

B

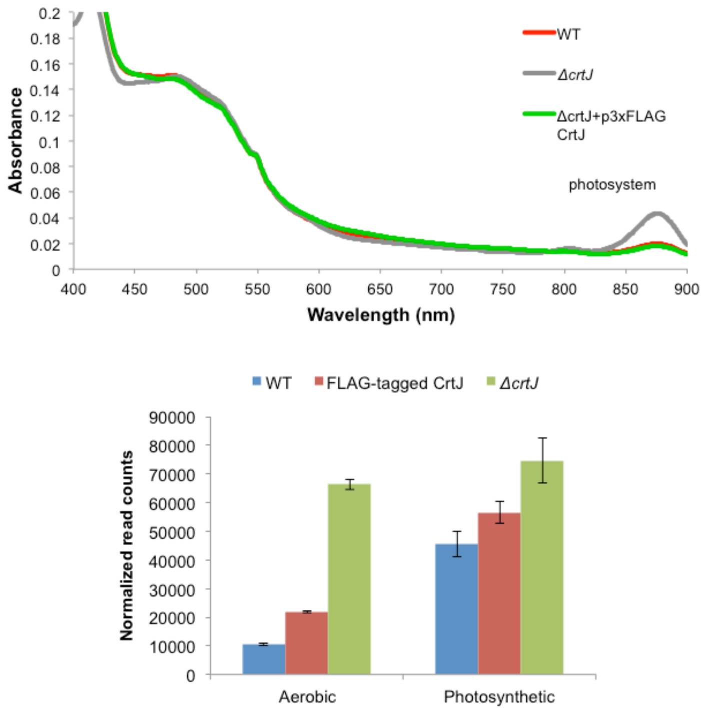

FCD Fig 2. A) Spectral analysis on different strain. B) Expression level of *bchC* gene in different strain based on normalized read counts from RNA-seq.

AerR functions as an activator of photosystem under photosynthetic condition, loss of it function leads to reduced production of photosystem [1]. To examine the activity of AerR-3xFLAG, the cells were grown under photosynthetic condition and the absorption spectra were compared among strains. Δ*aerR* strain showed a significant reduced pigment production compared to wild type, however, Δ*aerR* pSRKGm::*aerR*-3xFLAG showed comparable pigment production as wild type strain. This result suggested that AerR-3xFLAG still retains its function to activate photosystem.

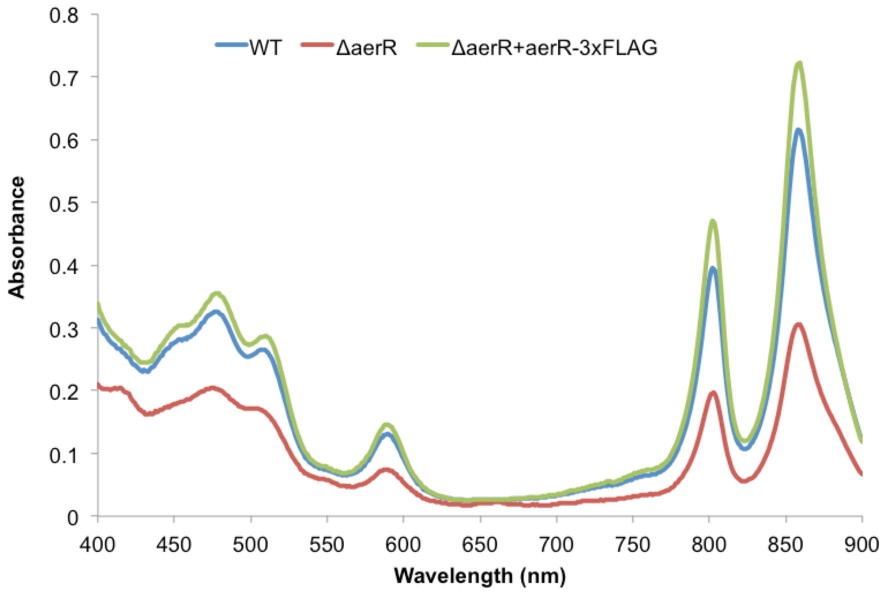

FCD Fig 3. Spectral analysis showing 3xFLAG-tagged AerR can rescue the phenotype ofΔ*aerR* strain.

Reference

1 Cheng Z, Li K, Hammad LA, Karty JA, Bauer CE (2014) Vitamin B12 regulates photosystem gene expression via the CrtJ antirepressor AerR in Rhodobacter capsulatus. Mol Microbiol 91: 649-664.
